# Unraveling transcriptome dynamics in human spermatogenesis

**DOI:** 10.1242/dev.152413

**Published:** 2017-10-15

**Authors:** Sabrina Z. Jan, Tinke L. Vormer, Aldo Jongejan, Michael D. Röling, Sherman J. Silber, Dirk G. de Rooij, Geert Hamer, Sjoerd Repping, Ans M. M. van Pelt

**Affiliations:** 1 Center for Reproductive Medicine, Amsterdam Research Institute Reproduction and Development, Academic Medical Center, University of Amsterdam, 1105 AZ Amsterdam, The Netherlands; 2 Bioinformatics Laboratory, Department of Clinical Epidemiology, Biostatistics and Bioinformatics, Academic Medical Center, Amsterdam, The Netherlands; 3 The Infertility Center of St. Louis, St. Luke's Hospital, St. Louis, Missouri, USA

**Keywords:** Gamete development, RNA-binding proteins, RNA-sequencing, Human, Spermatogenesis

## Abstract

Spermatogenesis is a dynamic developmental process that includes stem cell proliferation and differentiation, meiotic cell divisions and extreme chromatin condensation. Although studied in mice, the molecular control of human spermatogenesis is largely unknown. Here, we developed a protocol that enables next-generation sequencing of RNA obtained from pools of 500 individually laser-capture microdissected cells of specific germ cell subtypes from fixed human testis samples. Transcriptomic analyses of these successive germ cell subtypes reveals dynamic transcription of over 4000 genes during human spermatogenesis. At the same time, many of the genes encoding for well-established meiotic and post-meiotic proteins are already present in the pre-meiotic phase. Furthermore, we found significant cell type-specific expression of post-transcriptional regulators, including expression of 110 RNA-binding proteins and 137 long non-coding RNAs, most of them previously not linked to spermatogenesis. Together, these data suggest that the transcriptome of precursor cells already contains the genes necessary for cellular differentiation and that timely translation controlled by post-transcriptional regulators is crucial for normal development. These established transcriptomes provide a reference catalog for further detailed studies on human spermatogenesis and spermatogenic failure.

## Abstract (precis)

**Highlighted Article:** Using laser capture microscopy, a comprehensive transcriptomic dataset of well-defined and distinct germ cell subtypes based on morphology and localization in the human testis is generated.

## INTRODUCTION

Spermatogenesis is a tightly regulated developmental process during which mature haploid sperm cells are formed from diploid precursor spermatogonial stem cells. During this process male germ cells need to undergo strictly orchestrated complex cellular processes including mitotic divisions (pre-meiotic phase), meiotic divisions (meiotic phase) and extensive morphological transformations (post-meiotic phase). In each phase of human spermatogenesis, various developmental germ cell subtypes can be identified based on microscopic appearance and stages of the seminiferous epithelium (Amann, 2008; Muciaccia et al., 2013). In the pre-meiotic phase, A_dark_ (quiescent), A_pale_ (actively dividing) and B (differentiating) spermatogonia are present (Rowley et al., 1971; van Alphen et al., 1988). B spermatogonia eventually divide into pre-leptotene spermatocytes (de Rooij and Russell, 2000), which undergo the last round of DNA duplication prior to the meiotic divisions. In the meiotic phase, the most crucially important processes occur during the prophase of the first meiotic division: homologous chromosome alignment, meiotic recombination and crossover formation. After prophase I, spermatocytes progress through two consecutive meiotic divisions generating haploid round spermatids (Holstein et al., 1987). In the post-meiotic phase, round spermatids undergo extensive morphological changes, including chromatin compaction, reorganization of the cytoplasm and organelles, and development of the flagellum, eventually forming mature spermatozoa.

Disturbances in any of these phases can lead to spermatogenic failure. Various animal studies, as well as human cohort studies, have suggested a genetic basis for spermatogenic failure (Gurkan et al., 2013; Massart et al., 2012; Matzuk and Lamb, 2008; Sato et al., 2015; Song and Wilkinson, 2012; Stouffs et al., 2009; Venkatesh et al., 2014). Although these genetic studies provide useful information about spermatogenesis-related genes, they do not provide insights into the underlying molecular processes and as such cannot aid in understanding the pathobiology of spermatogenic failure and infertility.

In humans, knowledge about the molecular processes that control spermatogenesis is still limited. Recent gene expression studies have shown that human spermatogenesis is controlled by a complex network of molecular pathways (Bonache et al., 2014; Feig et al., 2007; Shafipour et al., 2014). However, these studies investigated expression changes in entire testicular biopsies from healthy and subfertile men. Owing to the highly heterogeneous mixture of cells present within the testis, these studies lack information on germ cell-specific expression and as such cannot pinpoint the molecular events required specifically for germ cell development. Furthermore, these studies have investigated gene panels selected beforehand and as such do not consider other potentially relevant genes not yet linked to spermatogenesis. Thus, generating germ cell-specific whole-transcriptome profiles is key to understanding the molecular landscape of each step during human spermatogenesis. Various groups have attempted to achieve this using various enrichment strategies; however, owing to the impurities of the isolated cell populations inherent to the techniques used, it has thus far been impossible to generate gene expression profiles of germ cell subtypes within the three phases during spermatogenesis (Chalmel et al., 2007; Zhu et al., 2016). For this purpose, we developed a protocol that allowed us to collect individual germ cell subtypes from fixed tissue using individual-cell laser-capture microdissection (LCM). Using this approach, we generated transcriptome profiles of six morphologically and functionally distinct successive germ cell subtypes that represent key steps in each phase of human spermatogenesis. Our data allow us to trace key gene expression dynamics during subsequent developmental steps throughout human spermatogenesis and furthermore provide a reference catalog for the characterization and understanding of various forms of spermatogenic failure and male contraception. Moreover, this study adds to the growing body of knowledge of processes involved in stem cell biology and cellular differentiation in other organ systems.

## RESULTS

Our primary aim was to generate a comprehensive transcriptomic dataset of well-defined and distinct germ cell subtypes present in the human testis using LCM. In itself this dataset, which is freely available to the scientific community (deposited in NCBI's Sequence Read Archive under accession number SRP069329), forms a basis for future studies and bioinformatics analyses on the etiology of spermatogenic failure, the development of male contraceptives and the basic understanding of human spermatogenesis. In the current paper we exemplify the usefulness of our dataset by addressing the following questions: What are the gene expression dynamics between successive germ cell subtypes during human spermatogenesis? What is the added value of germ cell subtypes compared with spermatogenic phases? How conserved is the germ cell transcriptome between human and mouse? How similar are A_pale_ and A_dark_ spermatogonia? What can we learn from post-transcriptional regulation in spermatogenesis by studying expression of RNA-binding genes and long non-coding RNA in the successive germ cell subtypes?

### LCM RNA-seq for transcriptome analyses of individual germ cell subtypes

**Fig. 1.**
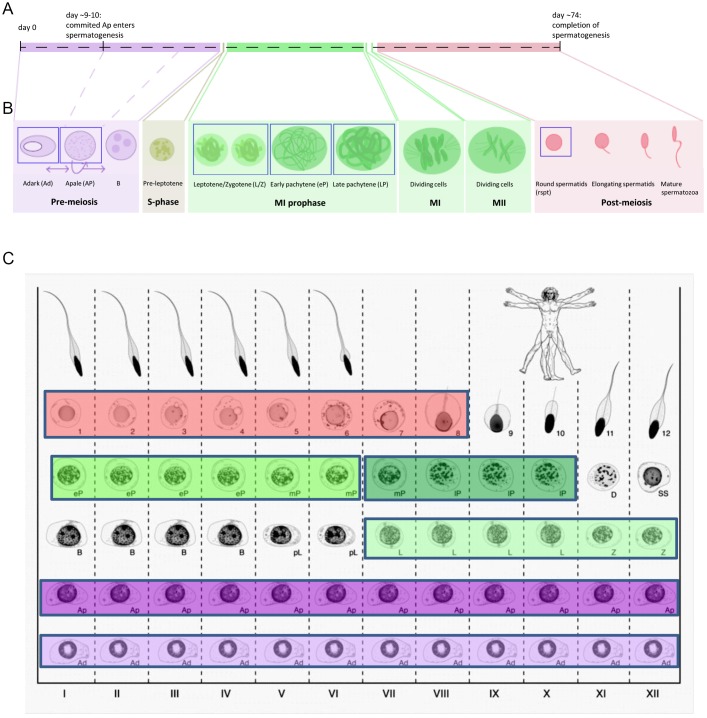
**Scheme of spermatogenesis and stages of the seminiferous epithelium in humans.** (A) A timeline showing the approximate relative time required for each developmental step of spermatogenesis. (B) Scheme of the various germ cell subtypes found within the testis. The germ cell subtypes collected for this study are boxed in blue. (C) An adapted scheme (Muciaccia et al., 2013) showing the stages of the seminiferous epithelium in man. Germ cell subtypes collected based on cellular association are depicted by the following colors: light purple (A_dark_ spermatogonia), dark purple (A_pale_ spermatogonia), pale green (leptotene/zygotene spermatocytes), bright green (early pachytene spermatocytes) and dark green (late pachytene spermatocytes).

First, we developed an LCM method that allows isolation of individual germ cell subtypes from fixed testicular tissue. Given the heterogeneity of germ cell subtypes within the testis that lie in close proximity to each other, we decided to use LCM to allow capturing of individual specific germ cell types while minimizing contamination with surrounding cells that would possibly be in a different stage of development. We first selected a fixative that maintains both RNA integrity as well as the typical morphological nuclear characteristics required to recognize individual germ cell subtypes (Fig. S1). Our developed method enabled recognition and selection of specific germ cell subtypes based on a combination of morphological criteria (Clermont, 1972), location in the seminiferous tubular cross-section and germ cell associations present in the various stages of the seminiferous epithelium (Muciaccia et al., 2013) within the three phases of spermatogenesis: pre-meiosis, meiosis and post-meiosis (Fig. 1). Five-hundred germ cells from each specific germ cell subtype were individually isolated using LCM and pooled. RNA from these pools was subsequently amplified and sequenced generating germ cell subtype-specific transcriptomic profiles. We extensively validated each individual step of this novel method (Figs S1 and S2). We demonstrate that this method allows for robust and reproducible amplification (r^2^=0.89) and sequencing of minute amounts of RNA from pools of 500 individually laser captured cells from 5-µm-thick sections using an alcohol-based fixative (see Materials and Methods; Figs S1 and S2). This method allowed us to capture specific germ cell subtypes, which cannot be isolated with any other cell sorting method, with high purity.

### Transcriptome profiles of spermatogenic phases

**Fig. 2.**
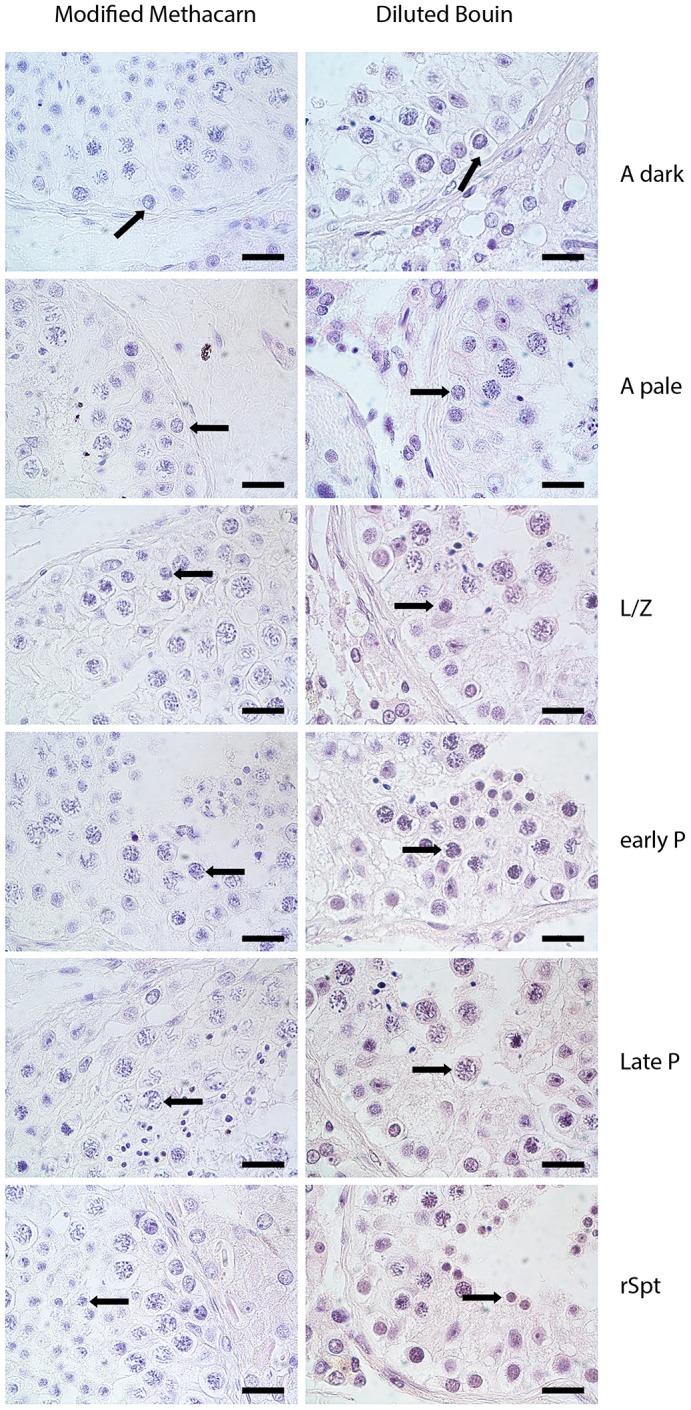
**Germ cell subtype identification.** Microscopic images showing the similarities in morphology of the germ cell subtypes in this study in testis tissue prepared using an alcohol-based fixative (modified methacarn) compared with tissue prepared using the standard fixative diluted Bouin's.

**Fig. 3.**
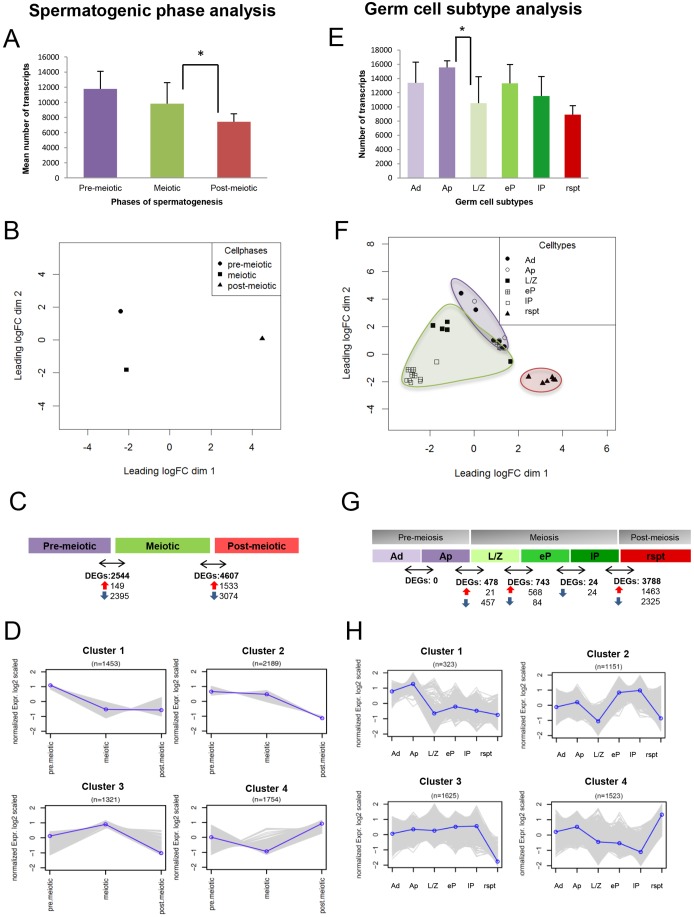
**Transcriptomic dynamics during spermatogenesis.** (A-D) Spermatogenic phase analysis of *in silico* pooled data representing the pre-meiotic (average of A_dark_ and A_pale_ spermatogonia, *n*=11), meiotic (average of leptotene/zygotene, early and late pachytene spermatocytes, *n*=17) and post-meiotic (round spermatids, *n*=6) phases of spermatogenesis from six patients. (A) Mean number of transcripts expressed based on counts per million (mean±s.d.) in the pre-meiotic, meiotic and post-meiotic phases. *P<0.05. (B) An MDS plot showing the transcriptomic variability between pre-meiotic, meiotic and post-meiotic samples. (C) The number of differentially expressed genes (DEGs) and their direction of change (red arrow, upregulated; blue arrow, downregulated) found between the different phases of spermatogenesis (adjusted *P*-value≤0.05). (D) K-means clustering of 6717 DEGs across spermatogenesis. (E-H) Germ cell subtype analysis of data (mean±s.d.) obtained from the germ cell subtypes: A_dark_ (*n*=6), A_pale_ (*n*=5) spermatogonia, leptotene/zygotene (*n*=5), early pachytene (*n*=6), late pachytene (*n*=6) spermatocytes, round spermatids (*n*=6). (E) Mean number of transcripts expressed in the germ cell subtypes. *P≤0.05. (F) An MDS plot showing the transcriptomic variability between the different germ cell subtypes: spermatogonia (purple; circles), spermatocytes (green; squares), spermatids (red; triangles). (G) Differentially expressed genes (red arrow, upregulated; blue arrow, downregulated) between the germ cell subtypes (adjusted *P*-value≤0.05). (H) K-means clustering of 4622 DEGs across spermatogenesis.

We were able to distinguish and select six specific germ cell subtypes (Fig. 2). We selected A_dark_ (Ad) and A_pale_ (Ap) spermatogonia to represent the pre-meiotic phase. As prophase I is the longest phase of meiosis, we chose three spermatocyte subtypes: leptotene/zygotene (L/Z), early pachytene (eP) and late pachytene (lP) to represent the meiotic phase. Lastly, we selected round spermatids (rspt) to represent the post-meiotic phase (Fig. 1). Using this protocol, we successfully determined the transcriptomes of these germ cell subtypes from six individuals with normal spermatogenesis [Table S1 (counts per million individual data) and Table S2 (averaged data from six men, mean±s.d.)]. Owing to very low numbers of B spermatogonia and pre-leptotene spermatocytes in human testicular tissue (Skakkebaek and Heller, 1973), the limited amount of available tissue and the need for at least 500 germ cell sections per individual germ cell subtype for our protocol, we were unable to include these germ cell subtypes for our RNA-seq analysis. We also decided to exclude elongating spermatids from this study because transcription is known to be silenced during DNA compaction in elongating spermatids. To be able to compare and validate our results with previously described data on spermatogenic phases, we first grouped our spermatogonial (A_dark_ and A_pale_ spermatogonia), spermatocyte (leptotene/zygotene, early pachytene or late pachytene) and spermatid data sets *in silico* to represent the major phases in spermatogenesis: pre-meiotic, meiotic and post-meiotic phases, respectively (Fig. 3A-D). Analysis of the mean number of expressed transcripts in each phase of spermatogenesis revealed that the pre-meiotic phase expressed the broadest gene set (mean of approximately 12,000 transcripts), which decreased by 17% (*P*=0.055) in the meiotic phase and further decreased by 24% (*P*=0.006) in the post-meiotic phase of spermatogenesis (Fig. 3A). The relative very complex transcriptome of the testis with respect to other organs has previously been reported and was explained by the complex transcriptome of spermatocytes and spermatids (Soumillon et al., 2013). However, we are the first to report a decrease in transcriptomic complexity as spermatogenesis proceeds with spermatogonia showing the highest complexity when investigating protein-coding genes on all chromosomes with exclusion of ribosomal genes. Interestingly, a decrease in transcriptomic complexity also occurs during cellular development in the lung epithelium (Treutlein et al., 2014).

**Fig. 4.**
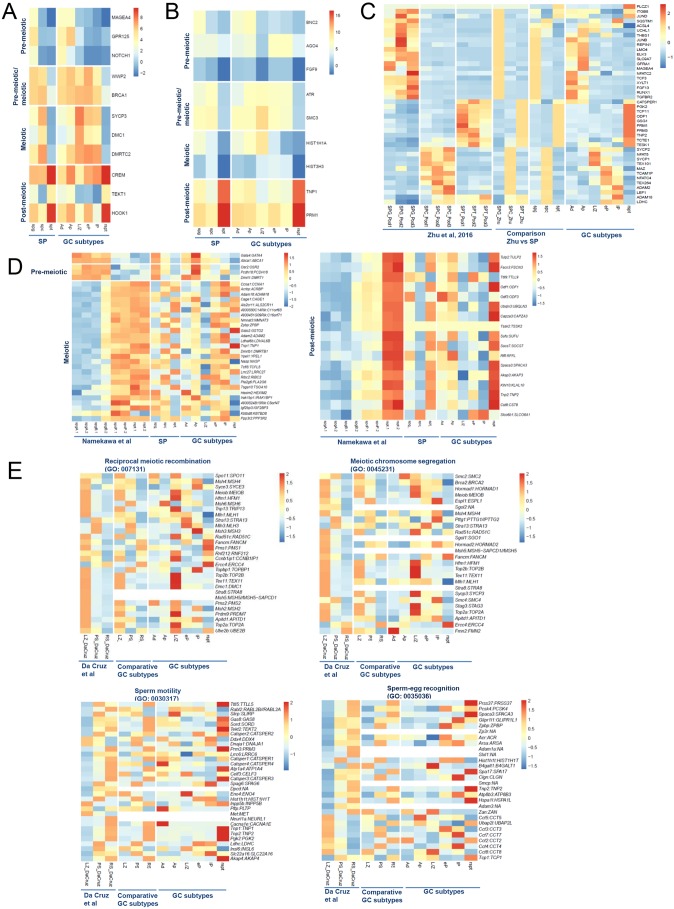
**Expression levels of selected pre-meiotic, meiotic and post-meiotic genes in man and mouse.** (A,B) Heatmaps displaying the expression levels of genes involved in spermatogenic phases. (C) Expression levels of selected genes from previously described spermatogenic phases analyses of human spermatogonia, spermatocytes and spermatids (Zhu et al., 2016) compared with our spermatogenic phase (SP) and germ cell subtype (GC subtypes) data. (D,E) Heatmaps comparing our spermatogenic phase and germ cell subtype data with reported mouse data on spermatogonia (spg), spermatocytes (spc) and spermatids (spt) (Namekawa et al., 2006) (D) and leptotene/zygotene spermatocytes (LZ), pachytene spermatocytes (PS) and round spermatids (RS) (da Cruz et al., 2016) (E).

Visualizing the mean of the transcriptomes of each individual germ cell subtype within each phase using multidimensional scaling (MDS) (Fig. 3B) revealed that germ cells of the pre-meiotic, meiotic and post-meiotic phases all have a distinct transcriptional fingerprint (Fig. 3B). We found 2544 differentially expressed genes (DEGs) between the pre-meiotic and meiotic phases and 4607 DEGs between the meiotic and post-meiotic phases of spermatogenesis (Fig. 3C). To enable visualization of expression dynamics of these DEGs, we used k-means clustering to group genes based on common expression patterns throughout spermatogenesis (Fig. 3D). Cluster 1 contains 1453 genes that are downregulated in the meiotic and post-meiotic phases compared with the pre-meiotic phase and includes well known spermatogonial genes associated with cell development and differentiation such as *NOTCH1*, *GPR125* (*ADGRA3*) and *MAGEA4* (He et al., 2010; Mori et al., 2003; Oatley et al., 2011; Sachs et al., 2014) (Fig. 4A; Tables S3 and S4), which is in line with the stem cell nature and propagation features of cells in the pre-meiotic phase. *ID4*, another spermatogonial marker (Sachs et al., 2014), was also observed more prominently in the pre-meiotic phase; however, owing to the large biological variation, the difference did not reach statistical significance (adjusted *P*-value=0.057). Cluster 2 contains 2189 genes, which are specifically downregulated in the post-meiotic phase relative to the other phases. This cluster is enriched with genes involved in protein ubiquitylation, DNA repair and transcription, such as *WWP2*, *BRCA1* and various genes encoding zinc finger proteins, all of which are processes that occur during the first two phases of spermatogenesis (Fig. 4A; Tables S3 and S4). Cluster 3 contains 1321 genes, which are upregulated in the meiotic phase relative to the pre-meiotic and post-meiotic phase, including genes like *SYCP3*, *DMC1* and *DMRTC2* (Bishop et al., 1992; Date et al., 2012; Kawamata et al., 2007; Lammers et al., 1994; Ozaki et al., 2011) involved in meiotic processes such as DNA repair and synapsis (Fig. 4A; Tables S3 and S4). Cluster 4 contains 1754 genes, which are upregulated in the post-meiotic phase compared with the other two phases and included genes involved in spermiogenesis such as *CREM*, *TEKT1* and *HOOK1* (Larsson et al., 2000; Mendoza-Lujambio et al., 2002; Weinbauer et al., 1998) (Fig. 4A; Tables S3 and S4). These results confirmed that our novel isolation procedure was able to isolate highly enriched populations of human germ cells.

### Transcriptome profiles of germ cell subtypes

Next, we performed a germ cell subtype analysis comparing the changing transcriptomic profiles of consecutive developmental germ cell subtypes within and between each phase of spermatogenesis (Fig. 3E-H; Table S1). This revealed that the gradual decrease in transcriptomic complexity during spermatogenesis as seen in Fig. 3A, is initiated by a significant drop in transcript number during early meiosis [comparing the dividing uncommitted A_pale_ spermatogonia with leptotene/zygotene spermatocytes (*P*≤0.0001)] (Fig. 3E). This is consistent with earlier studies describing low RNA production in leptotene/zygotene spermatocytes in mice (Monesi, 1964). This downregulation is then followed by an increase in the number of transcripts in early pachytene, and subsequent gradual decrease in number of transcripts upon completion of spermatogenesis. Investigating the distribution of these expressed transcripts reveals similar distribution across the chromosomes in all of the germ cell subtypes (Fig. S3).

MDS analysis confirmed that, although some biological variation within each phase was present, distinct clusters were visible for A_dark_ and A_pale_ spermatogonia, leptotene/zygotene spermatocytes, early and late pachytene spermatocytes, and round spermatids (Fig. 3F; Fig. S4).

### Germ cell subtype gene expression dynamics during human spermatogenesis

DEG analysis between germ cell subtypes revealed that the majority of gene expression changes occur during three steps: meiotic initiation (A_pale_ spermatogonia to leptotene/zygotene spermatocytes), homologous chromosome synapsis (leptotene/zygotene to early pachytene spermatocytes) and the start of spermiogenesis (late pachytene spermatocyte to round spermatids) (Fig. 3G). K-means clustering of all DEGs at successive developmental steps during spermatogenesis again revealed four major gene expression clusters (Fig. 4H; Table S5). Gene ontology analysis revealed that cluster 1 contains 323 genes that are highly expressed in A_dark_ and A_pale_ spermatogonia, which are related to transcription, steroid metabolism and cell differentiation. Surprisingly, we could not find a gene cluster with low expression in spermatogonia and high in all other germ cells. When analyzing 25 sub-clusters (Fig. S5), we found one cluster (cluster 9) with low expression in A_dark_ and A_pale_ spermatogonia and high in L/Z, eP and lP spermatocytes, followed by a decrease in round spermatids. This sub-cluster contains 141 genes of the total 4622 genes that are typically known to be meiosis related. Cluster 2 (Fig. 3H), containing 1151 genes that are downregulated in leptotene/zygotene spermatocytes and subsequently show high expression later during meiosis, which are related to spermatogenesis, mitosis, proteolysis and sperm-egg interactions (Tables S5 and S6). These two clusters contain genes that are crucial in regulating exit of the mitotic phase and entry into the meiotic phase such as *BNC2*, *AGO4* and *FGF9* (Fig. 4B; Tables S5 and S6). In line with our results, *Bnc2* and *Ago4* knockout mouse models have shown that mutant testes display premature expression of meiotic-associated proteins such as SYCP3, which leads to premature meiotic entry and subsequent meiotic arrest (Modzelewski et al., 2012; Vanhoutteghem et al., 2014). Similarly, FGF9 treatment of mouse spermatogonia leads to diminished numbers of meiotic cells and maintenance of the meiotic repressor *Nanos2* (Barrios et al., 2010; Bowles et al., 2010). We found 1474 genes that follow similar expression patterns during the switch from mitosis to meiosis (Table S5). These genes are all of interest when investigating spermatogenic failure that phenotypically result in a meiotic arrest.

We found the highest number of DEGs upon the onset of spermiogenesis (late pachytene to round spermatid) (Fig. 3G,H). Gene ontology analysis revealed that cluster 3 was enriched for genes related to mitotic and meiotic processes such as *SMC3*, *ATR* and genes encoding synaptonemal complex proteins, which were all downregulated in round spermatids (Table S6). In addition, in line with the histone-to-protamine transition occurring in spermatids, this cluster shows downregulation of various histone genes such as *HIST1H1A* and *HIST3H3.* Conversely, cluster 4 contains genes that are upregulated and required for histone replacement such as *TNP1*, *PRM1* and *PRM2* (Fig. 4B; Tables S5 and S6). This suggests that the switch from a late meiotic cell to a haploid cell requires dramatic gene expression changes that may be instrumental in the progression of and completion of meiosis and the initiation of sperm maturation.

### Comparing the transcriptome of germ cell subtypes with spermatogenic phases

With our RNA-seq analysis of specific germ cell subtypes within the three phases of spermatogenesis, we can now uncover the tightly regulated molecular control of human spermatogenesis. For example, the expression pattern of *AGO4* was not differentially expressed between the pre-meiotic and meiotic phase (Fig. 3B). However, in our germ cell subtype analysis, we can clearly see that on average *AGO4* is downregulated specifically in leptotene/zygotene spermatocytes compared with A_pale_ spermatogonia (adjusted *P*-value=0.09) and early pachytene spermatocytes (adjusted *P*-value=0.013). Although the former did not reach statistical significance (Table S1), we did detect statistically significant upregulation of *AGO4* expression in early pachytene spermatocytes. The dynamic expression pattern of *AGO4* during early meiosis suggests a possible dual function during the early mitotic-meiotic transition and later in meiosis. This information cannot be gained from mouse knockout studies or from crude expression analysis of the three spermatogenic phases. In other examples of the spermatogenic phase analysis, the expression of *DMC1*, *SYCP3* and *SMC3* (Fig. 4A,B), all involved in meiotic recombination and pairing and synapsis of the meiotic chromosomes, were highly expressed in the meiotic phase. However, by applying the germ cell subtype analysis, we found that these genes are significantly upregulated in leptotene/zygotene spermatocytes, i.e. during the early onset of meiosis. Exemplary is also the analysis of *HIST1H1A* (Fig. 4B), a gene necessary for compaction of chromatin. In the spermatogenic phase analysis, *HIST1H1A* expression was found mainly in the pre-meiotic phase, whereas at the germ cell subtype level, the expression was mainly localized in spermatogonia and leptotene/zygotene spermatocytes and not in early and late pachytene spermatocytes. Hence, our novel germ cell subtype data set provides crucial information about expression patterns otherwise lost when analyzed at the spermatogenic phase level.

Comparing our data set with the described specific gene expression set for human spermatogonia, spermatocytes and spermatids isolated by fluorescence-activated cell sorting (FACS) and magnetic-activated cell sorting (MACS) (Zhu et al., 2016), revealed that of the 66 genes detected by Zhu et al., 42 appeared differentially expressed in our data set and show a high similarity in expression profile at the specific spermatogenic phases (Fig. 4C; Fig. S6). More interesting, in our data set, genes could be further classified in very specific germ cell subtypes, which could not be done using the reported FACS method (Zhu et al., 2016). We found expression of the well-known spermatogonial genes *GFRA1* and *MAGEA4* in both A_dark_ and A_pale_ spermatogonia and the highest expression of the meiotic genes *SYCP1*, *SYCP2* and *NFAT5* in leptotene/zygotene spermatocytes. The SYCP proteins are components of the synaptonemal complex, but the role of NFAT5 in this germ cell subtype is unknown. In other cell types, NFAT5 is necessary for cell growth/volume and osmolarity (Ho, 2003). Knowing that the cell size of spermatocytes increases considerably, NFAT5 might have a function in this process. The spermatocyte genes *MAZ*, *TCAM1P*, *NFATC4* and *TEX264* were all mainly expressed in early pachytene spermatocytes. Whereas *ADAM2* and *ADAM18* were specifically expressed in late pachytene spermatocytes, *LEF1* and *LDHC* were expressed in both early and late spermatocytes. Furthermore, although the previously reported data set (Zhu et al., 2016) originated from three pooled samples of five men for spermatogonia, spermatocytes and spermatids, there is much heterogeneity between the three samples of each germ cell type. Our current results clearly indicate that this heterogeneity reflects variation in the developmental stages of the germ cells in each germ cell sample. Our method using morphology and testicular localization enables isolation of a higher degree of well-defined and distinct germ cell subtypes with less contamination and thereby a higher resolution in transcriptome profiles throughout germ cell development than, for instance, with cell sorting of germ cells by STA-PUT, elutriation, FACS or MACS. Because we could discriminate the transcriptomic dynamics in smaller developmental steps, the molecular mechanisms of human spermatogenesis could be unraveled in more detail.

### Comparing male germ cell transcriptomes between human and mouse

We investigated whether the male germ cell transcriptomes are conserved among species and compared our data set with available published data sets of mouse on spermatogenic phases (Namekawa et al., 2006) and on germ cell subtypes (leptotene/zygotene, pachytene spermatocytes and spermatids) (da Cruz et al., 2016), and used the categories and labels from these previously reported data sets in our comparative analyses (Fig. 4D,E), respectively. The expression of the three described pre-meiotic genes *Osr2*, *Pcdh18* and *Dmrt1* (Fig. 4D) in the mouse spermatogenic phases data set (Namekawa et al., 2006) are indeed well conserved in spermatogonia between human and mouse. Interestingly, the mouse meiotic gene set of pachytene spermatocytes, identified by microarray analyses, showed overall high expression in the human meiotic phase. However, in the germ cell subtype analyses, the mentioned genes showed low expression in human leptotene/zygotene spermatocytes and high in early and late pachytene spermatocytes, suggesting that the STA-PUT-isolated mouse spermatocytes mainly contain pachytene spermatocytes. The mentioned post-meiotic gene set was also well conserved between mouse and human.

More recently, da Cruz and colleagues determined the mouse transcriptome by RNA-seq from leptotene/zygotene, pachytene spermatocytes and spermatids isolated by FACS (da Cruz et al., 2016). Although they did not include a pure fraction of spermatogonia, we could compare our data set with their mouse gene set on the described gene ontology terms GO:007131 reciprocal meiotic recombination, GO:0045231 meiotic chromosome segregation, GO:0030317 sperm motility and GO:0035036 sperm-egg recognition (Fig. 4E). The genes in the GO terms reciprocal meiotic recombination and meiotic chromosome segregation are highly conserved in mouse and human and are all mainly expressed in leptotene/zygotene spermatocytes. Also the gene sets for sperm motility and sperm egg recognition are well conserved between mouse and human and mainly expressed by spermatids and/or pachytene spermatocytes. Because the mouse germ cells in this study were isolated from the first wave of spermatogenesis at ages 10-11 days postpartum (dpp) and 24-25 dpp, the germ cell-specific subtypes leptotene/zygotene and pachytene spermatocytes could be isolated based on the 4C DNA content at the two age ranges in mouse, respectively. Our method enables further discrimination among the pachytene spermatocytes into early and late pachytene spermatocytes, also in adults with complete spermatogenesis. Apart from similarities between mice and humans, we also found a clear difference in expression in the CATSPER gene family. The CATSPER proteins (CATSPER1, 2, 3 and 4) got lots of attention due to their proven role in ensuring sperm motility and thereby male fertility (Visser et al., 2011). Their exclusive expression in the testis have made them ideal target candidates for male contraceptives. For drug developers it is useful to know at what stage such genes are expressed for targeting purposes. In light of this, expression of *Catsper1*, *2*, *3* and *4* has been thoroughly investigated in mice (Li et al., 2007), but in humans only in ejaculated spermatozoa. Human ejaculated spermatozoa express *CATSPER1*, *2* and *3* but do not contain *CATSPER4*, whereas in mouse spermatozoa all four Catsper members are expressed. On the other hand, expression of all CATSPER members is described in human testis (Lobley et al., 2003), but it is not known in which cell types. We now demonstrate that *CATSPER1*, *2* and *3* are mainly expressed in round spermatids, whereas *CATSPER4* is mainly detected in spermatogonia (Fig. 4E) suggesting a function for CATSPER4 earlier in spermatogenesis compared with the other CATSPER family members. This information could be very useful in the field of male contraceptives.

### Quiescent and active spermatogonia have similar transcriptomic profiles

In non-human primates, the quiescent A_dark_ spermatogonia can be immediately activated upon depletion of the active A_pale_ population, as described after treatment with ionizing radiation (van Alphen et al., 1988). Interestingly, we did not observe any statistically significant DEGs between these two populations of spermatogonia (Fig. 3G). This implies that the proposed quiescent A_dark_ spermatogonia are virtually identical on a transcriptome level to A_pale_ spermatogonia and suggests that quiescent A_dark_ spermatogonia can rapidly switch to an active state while maintaining similar gene expression patterns. The variation observed within the A_dark_ and A_pale_ populations, is larger than that seen for the other germ cell subtypes from samples from the same men suggesting the variation is spermatogonial rather than individual specific (Fig. 3F; Fig. S4). This could explain the lack of DEGs found but also suggests that uncommitted cells are biologically more diverse than their differentiated counterparts. Despite this large variation, we see A_dark_ and A_pale_ clustering together (Fig. 3F) indicating these germ cell subtypes express a similar gene set. There have also been speculations regarding the presence of various spermatogonial germ cell subpopulations within both the A_dark_ and A_pale_ populations, which to date cannot be distinguished by morphological criteria. In support of this, it has recently been described that, at the single-cell level, human spermatogonia (without distinguishing between A_dark_ and A_pale_) display very diverse gene expression profiles (Neuhaus et al., 2017). To explore the possibility of subtle changes in molecular pathways between A_dark_ and A_pale_ spermatogonia, while accounting for inter-gene correlations, we used CAMERA (correlation-adjusted mean rank) analysis (Wu and Smyth, 2012). We found that one curated gene set, involving the neurotransmitter pathway, was significantly downregulated in A_pale_ compared with A_dark_ spermatogonia (non-adjusted *P*<0.01) (Table S7). Two genes were categorized within this pathway; namely, the glutamic acid decarboxylase known as *GAD1* and an amino acid hydroxylase known as *TPH1*. Both genes have been shown to play a role in the regulation of stem cell proliferation (Du et al., 2013; Ye et al., 2014). GAD1 is an enzyme involved in the synthesis of GABA receptors and has been shown to play a role in negatively regulating spermatogonial stem cell proliferation (Du et al., 2013). Its expression in A_dark_ spermatogonia may thus contribute to the quiescent state of these spermatogonia. On the other hand, TPH1, a mediator of serotonin synthesis, has shown to be expressed by human sperm (Jimenez-Trejo et al., 2012) but was not described in spermatogonia. We confirmed the expression of TPH1 in human spermatogonia at the protein level by immunohistochemistry (Fig. S7), suggesting that even if similar RNA expression levels are seen in A_dark_ and A_pale_, this would not exclude differences at the protein level. The use of proteomics in future studies might give more insights in potential differences between these two germ cell subtypes. In the hematopoietic system, TPH1-dependent serotonin depletion has been shown to negatively affect differentiation of erythroid precursors (Amireault et al., 2011) suggesting that perhaps TPH1 has a similar function in spermatogonia.

### Spermatogonia express (post)-meiotic genes

**Fig. 5.**
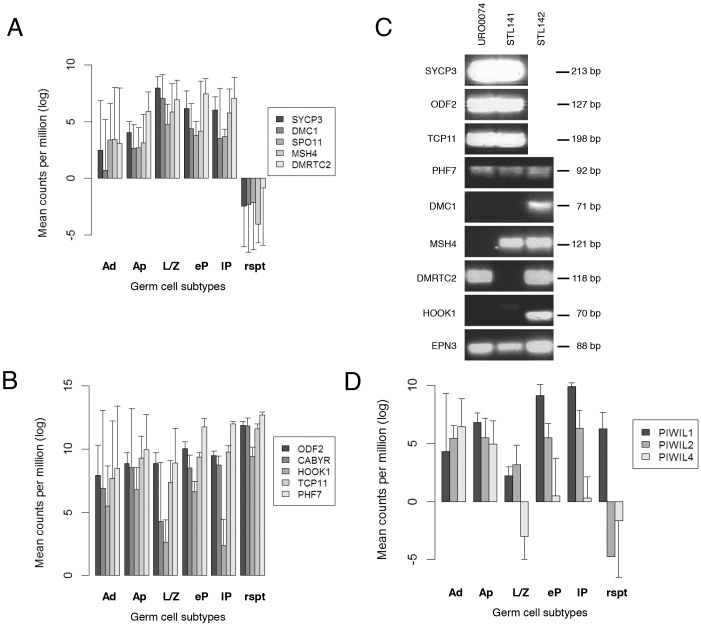
**Gene expression profiles of meiotic, post-meiotic and PIWIL genes.** (A,B) Bar charts showing expression levels represented by counts per million (log scale) (mean±s.d.) for meiotic (A) and post-meiotic (B) associated genes. (C) Expression measured by RT-PCR of four meiotic genes (*SYCP3*, *DMC1*, *MSH4* and *DMRTC2*), four post-meiotic genes (*ODF2*, *HOOK1*, *TCP11*, *PHF7*) and a reference gene (*EPN3*) in RNA isolated from spermatogonia collected from a spermatogonial arrest patient (URO0074; lane 1), and spermatogonia collected from vasectomy reversal men used for this study (Stl141 and Stl142, respectively; lanes 2 and 3). Note that owing to a limited amount of RNA from Stl142 we were unable to analyze *SYCP3*, *ODF2* and *TCP11*. (D) Bar chart showing expression level of genes (mean±s.d.) encoding the PIWIL proteins in each germ cell subtype during spermatogenesis.

Gene ontology analysis revealed that clusters 3 and 4 (Fig. 3H) are enriched with meiotic- and post-meiotic-associated genes. To our surprise, these genes all display low to moderate levels of gene expression in spermatogonia indicating that expression of (post-)meiotic-associated genes occurs during earlier steps in spermatogenesis. Indeed, further analysis confirmed that both A_dark_ and A_pale_ spermatogonia already express *SYCP3*, *DMC1*, *SPO11*, *MSH4* and *DMRTC2*, which are known to be functional during meiosis, and *ODF2*, *CABYR*, *HOOK1*, *TCP11*, *PHF7*, which are known to be functional in post-meiosis (Fig. 5A and 5B, respectively; Table S5). We confirmed these data with RT-PCR (Fig. 5C). To exclude the possibility that these findings are the result of cell impurities, we checked for the presence of eight of the aforementioned genes using RT-PCR. In the same way as for the RNA-seq experiments, we extracted RNA from 500 individually dissected and pooled spermatogonia from controls with complete spermatogenesis (Stl141 and Stl142) and from a man suffering from spermatogonial arrest (URO0074). Histological analysis revealed the complete absence of germ cells other than spermatogonia in patient URO0074 (Fig. S8). We confirmed the presence of five of the eight investigated genes (*SYCP3*, *DMRTC2*, *ODF2*, *TCP11* and *PHF7*) (Fig. 5C). *DMC1*, *MSH4* and *HOOK1* were undetectable in this sample but also showed variable expression in controls in which sequence counts were observed suggesting that the sensitivity of the RT-PCR technique might be insufficient to detect these genes. Consistent with our data, a study performed in chickens showed that transcripts of the meiotically associated SYCP family member homologs are already present in spermatogonia (Zheng et al., 2009) and many other transcribed genes are stored as translational inactive RNAs in ribonucleoprotein particles (Iguchi et al., 2006). The idea that genes encoding proteins required later during spermatogenesis are transcribed in spermatogonia is not new. Previously, studies in mice have indirectly suggested expression of meiotic and post-meiotic markers in pre-meiotic germ cells (Chalmel et al., 2012; Iguchi et al., 2006; Tanaka and Baba, 2005). In the present study, we now show expression of several specific meiotic- and post-meiotic-associated genes in human spermatogonia and have confirmed that these genes are not a result of contamination by other germ cell types. Together, these data suggest that spermatogonia express genes that are translated into protein and functional only later during differentiation.

### Expression of RNA-binding protein mRNAs

Consistent with the idea of post-transcriptional storage of mRNA, we found a number of common testis-specific RNA-binding proteins known to play a role in transposon silencing and more recently mRNA repression, storage and degradation such as the PIWI-like proteins (Bak et al., 2011; Vourekas et al., 2012), encoded by *PIWIL1*, *PIWIL2*, *PIWIL4*, which were all differentially expressed (adjusted *P*-value<0.05) across spermatogenesis in a germ cell subtype-specific manner. This suggested that these RNA-binding proteins play a role in controlling specific differentiation and developmental steps. In our study, *PIWIL1* expression fluctuated with highest expression levels in pachytene spermatocytes, suggesting a role for this protein during the meiotic phase (Fig. 5D). Consistent with our findings, recent *in vitro* protein interaction studies using recombinant PIWIL1 have shown that this protein can bind 3′UTR regulatory sequences of mRNAs encoding spermatid-specific proteins. Interestingly, these studies also showed that PIWIL1 can bind to protein and as such may play a role in post-transcriptional as well as post-translational regulation (Xu et al., 2014). Together with our expression data, these findings lead us to speculate that PIWIL1 plays a role in storing spermatid-specific mRNAs that are already expressed during the meiotic phase. In our data, *PIWIL2* was expressed at a constant level during the pre-meiotic and meiotic stages and decreased during the post-meiotic phase, whereas *PIWIL4* was expressed to a higher degree in the pre-meiotic phase relative to the meiotic and post-meiotic phases. Most studies on PIWIL2 and 4 focus on the transposon repressive functions of PIWI proteins (Carmell et al., 2007). However, considering that we find germ cell type-specific expression of these PIWI mRNAs, it would be interesting to investigate whether these two family members exhibit similar regulatory functions as PIWIL1.

**Fig. 6.**
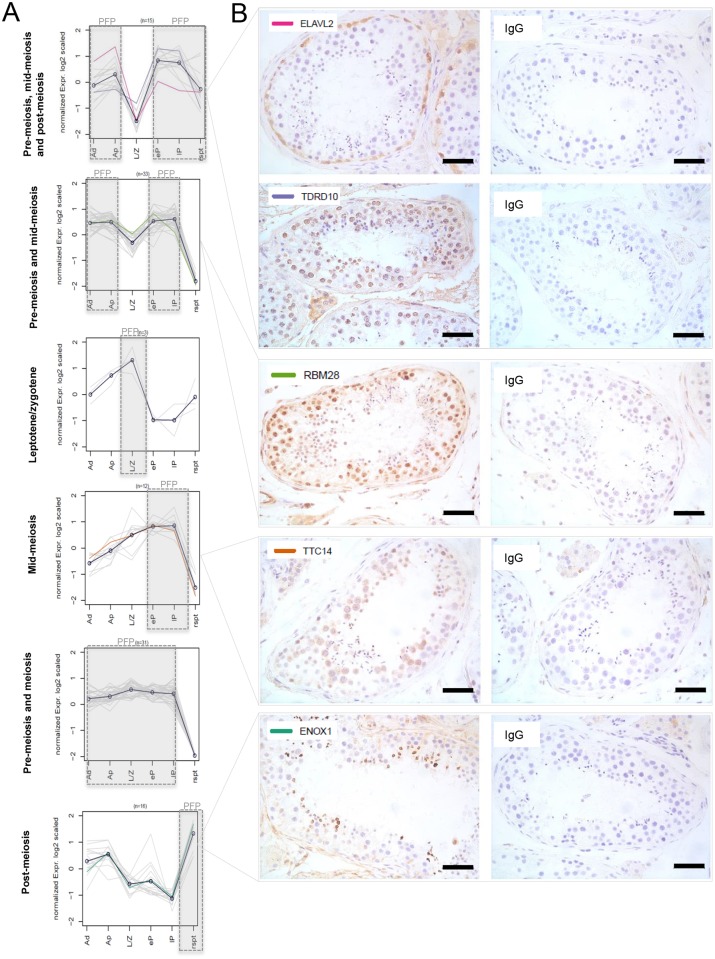
**Expression patterns of differentially expressed RNA-binding protein genes.** (A) K-means clusters showing the expression pattern of 110 differentially expressed RNA-binding proteins. The predicted functional phase is indicated by a gray box and ‘PFP’. (B) Immunohistochemistry of five selected RNA-binding proteins on testicular tissue of a prostate cancer patient (URO0368), repeated on three biopsies. Scale bars: 50 µm. Colored lines in the k-means clusters indicate the expression pattern of the mentioned corresponding gene.

Furthermore, we analyzed a list of RNA-binding proteins using a curated RNA-binding protein database (Cook et al., 2011) and found that 110 of the 416 curated genes in this database were significantly differentially expressed between germ cell subtypes (Table S8). Of these 110 RNA-binding proteins, 12 were known to play a role in post-transcriptional regulation during spermatogenesis, and 23 genes were known to be expressed in the testis without a known post-transcriptional regulatory function (Table 1). Interestingly, a recent study showed that the RNA-binding protein YBX2 was able to bind to the 3′UTR of elongated spermatid-specific mRNAs and loss of *YBX2* resulted in premature translation of these mRNAs (Cullinane et al., 2015). Indeed, we found that *YBX2* is expressed in early and late pachytene cells and even in round spermatids, in line with its function as a repressor of elongated spermatid-specific transcripts. In addition, we found 75 RNA-binding proteins previously not described during spermatogenesis. We therefore analyzed the gene expression profiles for these RNA-binding proteins, which resulted in gene clusters displaying six distinct expression patterns (Fig. 6). Based on these six clusters we proposed phases that we termed ‘predicted functional phases’ (PFPs) during which these RNA-binding proteins are highly expressed. In this phase we predict, based on high gene expression, that the gene is translated into protein and is functional. We further validated the results of our RNA-seq experiment by performing protein expression analysis using immunohistochemistry of five RNA-binding proteins (ELAVL2, TDRD10, RBM28, TTC14 and ENOX1) from four of the six K-means clusters (Fig. 6B). The protein expression of all five RNA-binding proteins was fully in line with the RNA-seq expression pattern within the germ cell subtypes and PFPs as presented in the respective K-means clusters (Fig. 6A). More precisely, ELAVL2 was expressed mainly in spermatogonia, TDRD10 was present in spermatogonia, early and late pachytene spermatocytes and its expression decreases in spermatids, RBM28 was present in all germ cells at relatively high levels except for spermatids, TTC14 was expressed mainly in early and late pachytene spermatocytes and ENOX1 was expressed only in round and elongating spermatids. Future functional studies can use this information to investigate post-transcriptional regulation during specific steps in spermatogenic development.

**Table 1.**
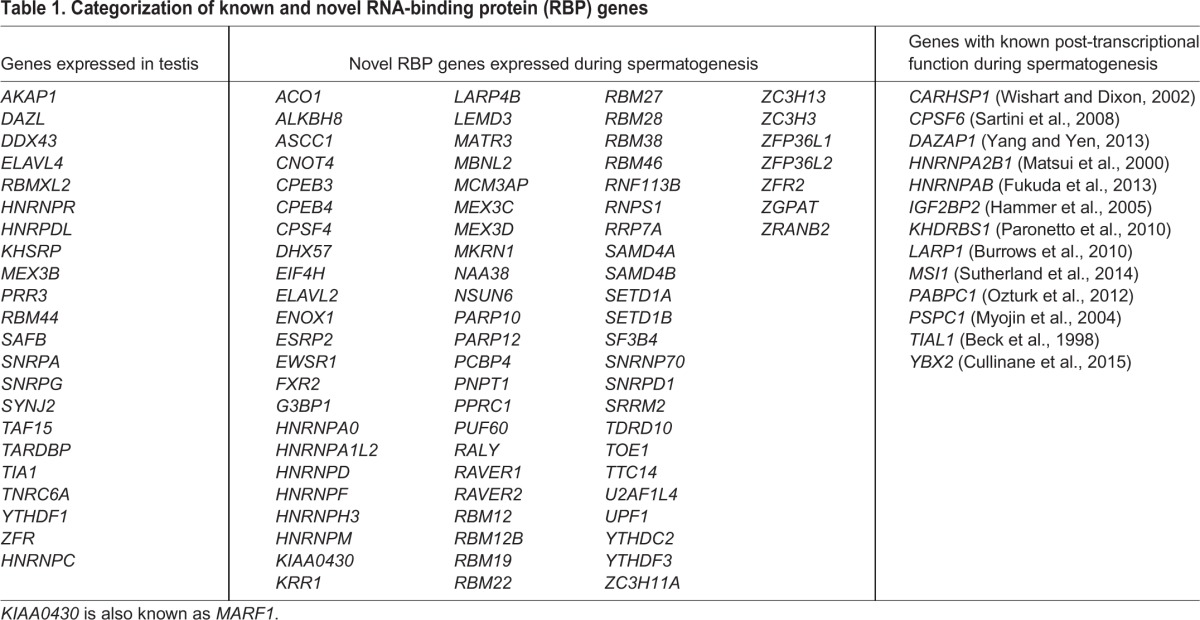
**Categorization of known and novel RNA-binding protein (RBP) genes**

### Differentially expressed long non-coding RNAs

Apart from RNA-binding proteins, long non-coding RNAs (lncRNAs) have also been proposed to play a major role in post-transcriptional gene regulation and chromatin remodeling (Yoon et al., 2013). We used a database provided by the HUGO Gene Nomenclature Committee at the European Bioinformatics Institute (Quek et al., 2015) to screen for lncRNAs in our dataset. We found 137 of the 2736 lncRNAs in this database to be differentially expressed during spermatogenesis (Table S9). These lncRNA genes also showed germ cell subtype-specific expression patterns, which once again implies that these genes play a role in controlling the various steps during spermatogenesis (Fig. S9).

## DISCUSSION

In this article, we describe the use of a novel protocol allowing for isolation, amplification and next generation sequencing of picogram amounts of RNA from specific germ cell subtypes collected from fixed human testicular tissue. This protocol could be useful for other studies involving complex tissues composed of a heterogeneous cellular population to determine cell type-specific transcriptomic profiles, but might also be useful for clinical studies where tissue and RNA is limited. In recent years, various techniques have been developed aiming to determine cell type-specific expression profiles of cells within a tissue. Tomo-seq (Junker et al., 2014) employs a combination of tissue section-specific barcoding and post-sampling reconstruction to determine expression profiles of particular regions within a tissue. Although a very elegant technique, it is limited to regional information and lacks individual-cell resolution and thus relies on the fact that in a certain region-specific cell types are found in bulk. This is not the case for human testicular tissue. Using other techniques, investigators have tried to improve resolution by using a combination of *in situ* hybridization alongside RNA sequencing of dissociated cells. These techniques rely on the availability of marker genes (stained by *in situ* hybridization) in order to reconstruct the expression profiles (Achim et al., 2015; Satija et al., 2015). To date no specific marker genes are known for the germ cell subtypes mentioned in this study. Furthermore, dissociation of the cells used in other methods such as STA-PUT, elutriation, FACS or MACS will result in loss of cellular localization characteristics thereby limiting the discrimination of various germ cell subtypes. To overcome such limitations, Lee and colleagues developed FISSEQ, a very sophisticated technique that allows for *in situ* RNA sequencing within fixed cells, thus maintaining cells in their *in vivo* environment (Lee et al., 2015). Although a very appealing technique, it has only been tested on rather simple model systems and the robustness with more complex heterogeneous tissues, as seen within the testis, is not yet known. Considering the limitations of these approaches, LCM is the preferable alternative that does not require post-sequencing spatial reconstruction and works well with simple as well as complex tissues. LCM comes with an added benefit of allowing selection of cell types of interest based on morphological criteria and/or location within a tissue (Nakamura et al., 2007). Our newly developed LCM protocol is unique as we, for the first time, report the use of LCM to dissect individual germ cell subtypes from testis tissue fixed in a way that keeps the necessary morphological typical nuclear germ cell characteristics intact, while maintaining sufficient RNA integrity for whole transcriptome profiling. Although LCM allows cell morphological and localization characteristics to specify germ cell subtypes that otherwise cannot be isolated with current other methods, the amount of RNA from cells obtained by LCM of 5-µm-thick slides is low and therefore requires at least 500 cell sections for reproducible results.

The lack of transcriptomes from specific germ cell subtypes has been a major obstacle for reproductive medicine and biological research. Using the newly developed protocol described in this article, we have generated more detailed human germ cell subtype-specific transcriptome profiles than has thus far been possible with any other method. In addition, fixing the tissue directly after tissue collection allows us to avoid *in vitro* handling of the cells, eliminating the effects of external influences on the cells prior to RNA isolation thereby representing as close as possible the *in vivo* RNA expression profile of these cells. Nevertheless, comparing our spermatogenic phase data with previous transcriptome data of FACS and MACS sorted human spermatogonia, spermatocytes and spermatids (Zhu et al., 2016) shows high similarities. Whereas they pooled tissue from 15 patients in three biological replicates for isolation of the three spermatogenic phases, we analyzed more specific germ cell subtypes from individual patients thereby creating the opportunity to study individual variations in transcriptomes, which will be useful when analyzing transcriptomes of patients with a developmental arrest in specific germ cell subtypes in spermatogenesis in future studies.

The most dramatic changes in gene expression occurred during early meiosis and during meiotic exit. Strikingly, the germ cell subtypes A_dark_ and A_pale_ spermatogonia are highly similar in transcriptome pattern implying that the switch from a quiescent to active cellular state is not controlled at the transcriptional level. Furthermore, a high number of transcripts known to be required at a protein level during the later phases is already present in spermatogonia. This implies that uncommitted cells already express, albeit at low levels, lineage-specific genes that are required during specific differentiation steps later on. It may be biologically more efficient for uncommitted cells to express genes required later during differentiation so that upon stimulation by external triggers they can rapidly start the differentiation process. This suggests that more primitive uncommitted cells house the building blocks required for differentiation into a specialized cell. Our data reveal that timely and highly regulated transcription and translation, regulated by RNA-binding proteins or lncRNAs, are crucial for cellular differentiation, a phenomenon that could be generalized to development of various tissues. Indeed, there is evidence in other tissues such as the lung, intestine and blood to support this notion (Tadjali et al., 2002; Treutlein et al., 2014; Yang et al., 2013).

When analyzing the entire process of spermatogenesis, we found the highest transcriptomic complexity before meiosis. Although having similar transcriptomic profiles, we see that the variation in gene expression levels within the A_dark_ and A_pale_ populations is larger than that seen for the other germ cell subtypes in samples from the same men. This suggests a heterogeneity within the spermatogonial populations that cannot be distinguished morphologically and is present within both the A_dark_ or A_pale_ spermatogonia. Indeed, the presence of two or more subpopulations among spermatogonia has recently been reported in various species including mouse (Hermann et al., 2015), rhesus monkey (Simorangkir et al., 2009) and human (Di Persio et al., 2017; Neuhaus et al., 2017).

Being able to assign a gene expression profile to the different germ cell subtypes is an important step forward in our understanding of the molecular control of human spermatogenesis. These profiles can be used in future comparisons with transcriptomic data from patients with arrested spermatogenesis to unravel the molecular causes of human spermatogenic failure. In addition, understanding how this developmental process occurs also enables us to understand how to inhibit spermatogenesis for developing male contraceptives. Besides this, the transcriptomic catalog helps us to understand key molecular events during cellular differentiation in the testis, which could be applicable to stem cell differentiation within other organ systems.

## MATERIALS AND METHODS

### Tissue collection

Testicular biopsies were routinely collected from six men undergoing vasectomy reversal procedures approved at The Infertility Center of St. Louis, USA (Stl) and during testicular sperm extraction (TESE) or orchiectomy as part of a prostate cancer treatment approved at the Academic Medical Center (Amsterdam, The Netherlands; URO). An important point to note is that the transcriptome profiles are generated from men who were undergoing a vasectomy reversal procedure. Histological analysis of all tissues used in our experiments indicated that all men had complete and normal spermatogenesis. Furthermore, all men had children before vasectomy. Mature sperm were measured and detected in post-vasectomy-reversal samples from 5/6 men and successful impregnation was recorded in 4/6 men including one individual in which sperm parameters were not measured. This confirms that the germ cell subtypes used to generate these transcriptome profiles are representative of normal intact and fully functional spermatogenesis. The ages of these men ranged from 38 to 58 years and all gave informed consent. The biopsies were fixed in modified methacarn (90% methanol, 10% glacial acetic acid) and embedded in paraffin because this showed optimal morphology of the testis while maintaining the highest RNA integrity. All procedures were performed under RNase-free conditions. All vasectomy reversal samples (Stl142, Stl141, Stl140, Stl138, Stl136, Stl135) and the prostate cancer sample (URO0368) used for this study had complete spermatogenesis and patient material from URO0074 had a spermatogonial arrest phenotype as confirmed by histological evaluation with Hematoxylin and Eosin staining.

### Selection of germ cell subtypes

Germ cell subtypes were identified based on morphological nuclear criteria according to Clermont (1972) and germ cell association in the chosen area of the seminiferous tubule (stages of the seminiferous epithelium) according to Muciaccia et al. (2013). A_dark_ and A_pale_ spermatogonia were selected based on morphological nuclear criteria and their localization on the basal membrane of the seminiferous tubules. A_dark_ spermatogonia were identified by a ‘donut-like’ dense chromatin in the nucleus characterized by a rarefaction zone in the center, whereas A_pale_ spermatogonia were identified by evenly distributed euchromatin in the nucleus surrounded by a thin dense border containing no more than one dense nucleolus. The different spermatocyte subtypes were selected based on nuclear staining and the association of germ cells in the chosen tubular segment, i.e. the stage of the epithelial cycle. Leptotene/zygotene spermatocytes (stages VII-XII) were identified as having condensed chromosomes in the nucleus and being located under (basal side) a second layer of larger late pachytene spermatocytes, which contained loose, less-dense, big chromosomes (stages VII-X). Early pachytene spermatocytes were identified as germ cells containing loose, less-dense chromosomes located in an area with only one layer of pachytene spermatocytes (stages I-VI) in association with round spermatids. Round spermatids were identified as small, round germ cells with a dense nuclear staining located at the luminal side of the tubule (Stages I-VIII).

### Individual-cell laser capture microdissection

Directly prior to laser capture microdissection (LCM), 5-µm-thick sections of fixed testis tissue were mounted on Superfrost glass microscope slides (Thermo Scientific) and stained using an adapted quick Hematoxylin and Eosin protocol for LCM (using short incubation time and RNase-free solutions). Immediately after staining, the tissue was covered with 1 µl of mineral oil, diluted 1:5 in isopropanol, and used immediately for LCM (inverted PALM microscope, Carl Zeiss) (Fig. S2). To minimize the risk of contamination, we used the smallest possible laser beam for LCM to capture 5-µm-thick sections of only the nucleus and cytoplasm closely surrounding the nucleus of the selected cells without prior cutting. Only those cells were captured in which the nucleus was clearly visible for morphological characteristics. We evaluated each potentially selected germ cell at various focal levels to ensure that only one type of germ cell was present in the selected area. To exclude the possibility of cross-contamination between germ cell subtypes during capturing, sections were exclusively used for capture of one specific germ cell subtype. For each cell type, new sections were prepared. We used a 400× magnification (40× objective and 10× ocular lens) on the laser capture microscope for dissection, confirmation and contamination check. We closely examined all sections before and after LCM to ensure that no additional cells other than those we aimed to capture were removed from the section. Samples showing signs of possible contamination were discarded. For each germ cell subtype, 500 cells were individually laser dissected and pooled according to subtype. Laser-dissected cells were captured in silicon coated adhesive caps (Adhesive cap 500 opaque tube, Zeiss) and were lysed at 42°C in 10 µl of extraction buffer provided in the PicoPure RNA isolation kit (Arcturus). Cell lysates were stored at −80°C until further use for RNA sequencing on the HiSeq2000 Illumina platform (supplementary Materials and Methods). All procedures were performed under RNase-free conditions.

### RNA integrity

Because RNA isolated from fixed tissue is usually partially degraded, we used the Paradise PLUS reagent system (protocol B) developed by Arcturus (Life Technologies). This is a method developed to determine RNA integrity of formalin-fixed paraffin-embedded (FFPE) samples. Briefly, this qPCR-based method uses an oligo dT primer and primers specific to a proximal and distal site of the 3′UTR of the β-actin gene to generate cDNA transcripts which are subsequently amplified. A ratio of proximal:distal transcript number gives an indication of RNA integrity/degradation. A ratio of 1 represents completely intact RNA and greater than 1 partially degraded RNA. The mean RNA integrity value measured in our samples was 2.13. According to manufacturer's instructions a value of <20 is acceptable for expression measurements by qPCR or RNA-seq.

### Bioinformatics

All sequence data have been submitted to NCBI (SRA) and are available under the accession number SRP069329. Raw reads were trimmed using Trimmomatic v32 and corrected using SEECER (Le et al., 2013). Trimmed reads were mapped to the hg19 human genome using TopHat/Bowtie2.0 using default parameters. Count data were obtained using the HTSeq program (version 0.5.4p3) using the UCSC hg19 GTF annotation file. Analyses were carried out with Bioconductor packages using the statistical software package R (version 3.1.2). Comparative analyses between the consecutive spermatogenic phases and consecutive germ cell subtypes were performed using the Voom method (Law et al., 2014). Differentially expressed genes were considered statistically significant when the adjusted *P*-value was ≤0.05 (see supplementary Materials and Methods for detailed individual bioinformatics analyses).

### RT-PCR

RT-PCR was performed on RNA isolated and amplified from 500 laser-dissected spermatogonia from a testicular biopsy from a spermatogonial arrest patient and from two individuals with complete spermatogenesis, which were also used in the sequencing study (Stl141 and Stl142). RT-PCR was performed on selected genes for validation of the RNA-seq data (see supplementary Materials and Methods for further details including primer sequences).

### Immunohistochemistry

Immunohistochemical staining was performed on 5-μm-thick paraffin human testis sections with or without antigen retrieval in sodium citrate, pH 6.0 at 98°C for 10 min. After inhibition of endogenous peroxidase by 0.3% H_2_O_2_, sections were blocked with Superblock (ScyTek). Subsequently, sections were incubated with rabbit primary antibody overnight at 4°C. Primary antibodies used were as follows: anti-ELAVL2 (1:500), anti-TTC14 (1:20) and anti-ENOX1 (1:200) (HPA063001, HPA009295 and HPA038355, respectively, all from Atlas Antibodies), anti-RBM28 (1:20) and anti-TDRD10 (1:50) (ab150800 and ab121982, respectively, both from Abcam) and anti-TPH1 (1:50) (OAAB04046, Avivia Systems Biology). After washing, the sections were incubated with biotin-conjugated anti-rabbit IgG (Vector Laboratories) and subsequently with Avidin-Biotin complex (ABC, Vector Laboratories) and visualized using 3,3′-diaminobenzidine (DAB, Alfa Aesar) as a brown substrate and Hematoxylin as counterstain. As negative controls, we used isotype rabbit IgG instead of the primary antibody. Slides were examined using a brightfield microscope (Olympus BX41).

## Supplementary Material

Supplementary information
