# Supplementary material for: Unraveling transcriptome dynamics in human spermatogenesis
Source: Development. 2017 Oct 15;144(20):3659–73. doi: 10.1242/dev.152413 (PMC5675447; doi:10.1242/dev.152413)
Supplement: Supplementary information [file develop-144-152413-s1.pdf]

SUPPLEMENTARY MATERIALS AND METHODS

RNA isolation, amplification and sequencing

Total RNA was isolated from cell lysates using the PicoPure RNA isolation kit (Arcturus) according to the manufacturers' protocol including an on-column DNase treatment. RNA was eluted in 10µl elution buffer. Subsequently, the RNA was concentrated to a volume of 5 µl with a speed vacuum centrifuge for 8 minutes.

Total RNA isolated from 500 pooled cell sections was amplified using the Ovation RNAseq V2 System (Nugen) according to the manufacturers' protocol. Reproducibility of the RNA amplification was confirmed using total RNA from three sets of 500 spermatocyte sections that were pooled after RNA isolation and split again into three technical replicates and subsequently amplified and sequenced.

For sequencing, the amplified SPIA-cDNA was sheared using the Covaris S220 (Thermo Fisher Scientific) with the following settings: DC 10%, I 5, C/B 200, T(s) 180. Sheared samples were visualized on the 2100 Bioanalyzer (Agilent Technologies). Insert sizes of ~200bp were selected using Agencourt AMPure beads (Beckman Coulter). DNA libraries were made using the DNA Truseq (Illumina) library preparation protocol and sequenced single-end, 50bps on the HiSeq2000 Illumina platform obtaining at least 10 million reads using 8 pmol per library.

Detailed bioinformatics

Count data was normalized using in edgeR (version 3.8.6) with default parameters (R version 3.1.2). Samples with a normalization factor between 0.6-1.4 were included in the analyses. One A_{pale} (Stl 135) and one leptotene/zygotene (Stl 136) sample was removed from the analyses since the normalization factor was outside the set criteria. To determine the number of

Development • Supplementary information

genes present in each phase of spermatogenesis, a gene was considered expressed in each germ cell subtype if it had >1 count per million present in at least three individuals. Raw counts were transformed to moderated log-counts-per-million before filtering using the cpm function with default parameters. For initial analyses of the transcriptome complexity of the phases of spermatogenesis, the various germ cell subtypes in each phase were grouped *in silico* as follows: pre-meiotic phase (mean number of transcripts expressed in spermatogonia; 6 A_{dark}, 5 A_{pale} samples), meiotic phase (mean number of transcripts expressed in spermatocytes; 5 leptotene/zygotene, 6 early pachytene and 6 late pachytene spermatocyte samples) and post-meiotic phase (mean number of transcripts expressed in spermatids; 6 round spermatid samples). Welch's t-test was used to test for statistically significant differences in the mean number of transcripts present. Genes were reannotated using *biomaRt* (version 2.22.0). The MDS plots are based on the 500 genes most relevant for distinguishing each pairwise comparison of samples. A list with differentially expressed genes (DEGs) between various germ cell types was obtained by estimating the mean-variance of the log counts using the *voom* method (Law et al.,2014) and analysing these with the empirical Bayes pipeline as implemented in *limma* (version 3.22.7) . After correcting for multiple testing, a p-value of <0.05 was considered significant for DEG analysis. K-means clustering (default algorithm) was used to obtain plots for the scaled normalized relative gene expression data on a log scale for each expressed gene using packages *clValid* (version 0.6-6), *cluster* (version 2.0.2) and *stats*. Gene ontology analysis was performed using the functional annotation clustering tool in DAVID. An enrichment score of >1.3 was considered significant (Huang da et al.,2009). To analyse gene sets, a more modest non-adjusted p-value of <0.01 was used for the Correlation-Adjusted MEan RAnk (CAMERA) gene set test (*limma*). CAMERA is a gene set test which determines differences in molecular control by

Development • Supplementary information

looking at sets of genes rather than individual DEGs, thereby accounting for inter-gene correlation (Wu and Smyth,2012). Gene sets were obtained from MSigDB (version 4.0, collections 2,3,5-7). For comparison of our RNA-seq data with previously described human and mouse germ cell RNA-seq data we used the RPKM values (reads per kilobase per million mapped reads) of the previously described selected gene sets (Zhu et al.,2016) (Supplementary File 2) and of the gene sets in the GO terms as described in another study (da Cruz et al.,2016). RPKM values were mean centred and scaled. For comparison of our data set with that of previously described selected mouse gene set (SI Fig 9a and b) we used mouse microarray data as previously described (Chalmel et al.,2007) from the reported Geo dataset: GSE4193 generated by Affymetrix microarray (Namekawa et al.,2006). Probes were annotated according to the Affymetrix Mouse 430 2.0 (GPL1261) annotation using the R package mouse430v2.db. Probes with the highest expression level were selected if multiple probes per gene existed. Expression values were log2 transformed, mean centered and scaled. For all comparisons with both mouse data, mouse gene names were mapped to the human orthologs using BioMaRt (release 65, www.ensembl.org) and Homologene (build 68, ftp://ftp.ncbi.nih.gov/pub/HomoloGene/) and only those genes that mapped with the human orthologs were used in this study. In case of previously described human data (Zhu et al.,2016) (Fig 3c), row clustering was applied when generating the final heatmap.

RT-PCR

RNA was amplified with the Nugen amplification kit and the resulting cDNA was used for SYBR green based (Roche) RT-PCR. Primer sequences used: *SYCP3* (tggaaactcagcagcaagagat / tgacttaacagaaagggaggtc), *DMC1*

Development • Supplementary information

(tgagatgcctgaaaatgaagcc/ccacctactccttggcatcc), *MSH4* (tggacaccacaagtgggata / ttgccattcctatttcacctc), *DMRTC2* (gaaccactcagccacaggtc / agaggcccacaggagttctt), *ODF2* (cgaaggcagttccagtctca / tgaggtcgatgttcttccgc), *HOOK1*(cctatttctttctagggcacttctg / cacagacactactgtggcctaaa), *TCP11* (atcagcaggtgtttggtccc / ggcctgggatagaagctcac), *PHF7* (agaaggaatgccagagattgag / aaggcatagccagcaaacag), *PFN3* (tatatttccccgagaggtgcg / tgatgtagaccttccagtcgc), *EPN2* (ttgtggacaggagcctcata / tcctttggagcctcagattc). Primers were designed using primer blast on the NCBI website. Primers were screened for SNPs and possible binding to pseudogenes. RT-PCRs with the selected primers were optimized and validated using RNA from whole testicular biopsies with a gradient PCR. Only primers that resulted in a single band on gel were used in the experiment. Amplicons (ranging from 70 to 200 bp) resulting from all primers were sequenced to confirm the primers amplified the target genes.

References

Chalmel, F., Rolland, A. D., Niederhauser-Wiederkehr, C., Chung, S. S., Demougin, P., Gattiker, A., Moore, J., Patard, J. J., Wolgemuth, D. J., Jegou, B. and Primig, M. (2007) The Conserved Transcriptome in Human and Rodent Male Gametogenesis. *Proc Natl Acad Sci U S A*, **104**(20), 8346-51.

da Cruz, I., Rodriguez-Casuriaga, R., Santinaque, F. F., Farias, J., Curti, G., Capoano, C. A., Folle, G. A., Benavente, R., Sotelo-Silveira, J. R. and Geisinger, A. (2016) Transcriptome Analysis of Highly Purified Mouse Spermatogenic Cell Populations: Gene Expression Signatures Switch from Meiotic-to Postmeiotic-Related Processes at Pachytene Stage. *BMC Genomics*, **17**, 294.

Huang da, W., Sherman, B. T. and Lempicki, R. A. (2009) Systematic and Integrative Analysis of Large Gene Lists Using David Bioinformatics Resources. *Nat Protoc*, **4**(1), 44-57.

Law, C. W., Chen, Y., Shi, W. and Smyth, G. K. (2014) Voom: Precision Weights Unlock Linear Model Analysis Tools for Rna-Seq Read Counts. *Genome Biol*, **15**(2), R29.

Namekawa, S. H., Park, P. J., Zhang, L. F., Shima, J. E., McCarrey, J. R., Griswold, M. D. and Lee, J. T. (2006) Postmeiotic Sex Chromatin in the Male Germline of Mice. *Curr Biol*, **16**(7), 660-7.

Wu, D. and Smyth, G. K. (2012) Camera: A Competitive Gene Set Test Accounting for Inter-Gene Correlation. *Nucleic Acids Res*, **40**(17), e133.

Zhu, Z., Li, C., Yang, S., Tian, R., Wang, J., Yuan, Q., Dong, H., He, Z., Wang, S. and Li, Z. (2016) Dynamics of the Transcriptome During Human Spermatogenesis: Predicting the Potential Key Genes Regulating Male Gametes Generation. *Sci Rep*, **6**, 19069.

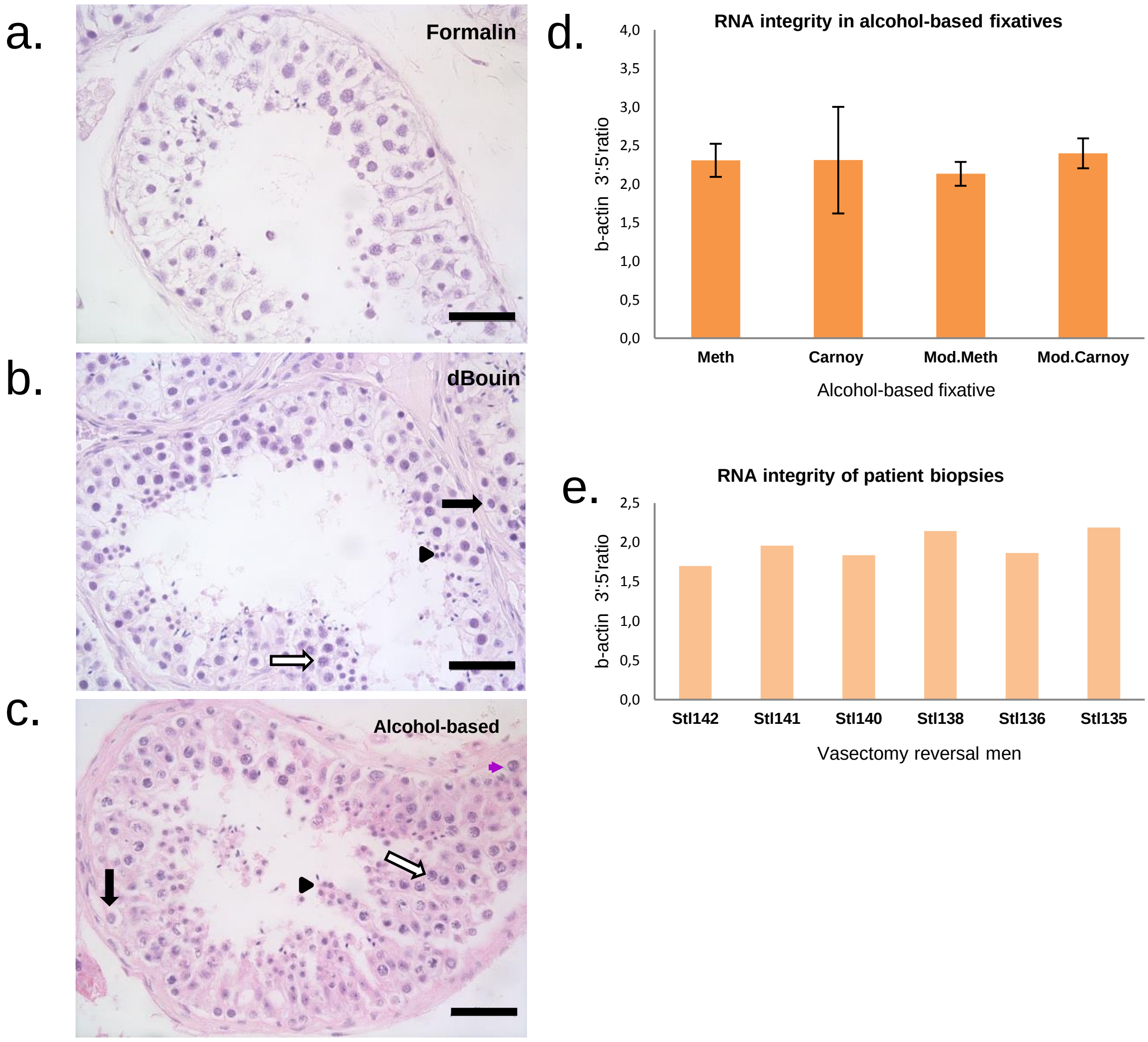

Figure S1. Selection of fixative.

Fixatives were selected to give the best morphology while maintaining RNA integrity. Poor morphology was observed with formalin fixation (a) and the best morhophology was observed with diluted Bouin (b) and alcohol-based fixatives (c). Black arrow: spermatogonia, open arrow: spermatocyte, and black arrowhead: spermatid; bar: 50µM. (d) RNA integrity was tested using the Paradise PLUS reagent system (protocol B) developed by Arcturus (Life Technologies), a qPCR based method uses oligo dT primers to generate cDNA transcripts which are subsequently amplified using primers specific to a proximal and distal portion of the 3'UTR of the b-actin gene. A ratio of proximal:distal transcript number gives an indication of RNA integrity. A ratio of 1 represents completely intact RNA and greater than 1 partially degraded RNA. According to manufacturer's instructions a value of <20 is acceptable for expression measurements. The RNA integrity in tissue fixed in four alcohol-based fixatives revealed that the lowest ratio (mean ±SD), was achieved with modified methacarn (n=3). (e) The RNA integrity values measured in our samples was approximately 2, indicating RNA of sufficient quality.

Development • Supplementary information

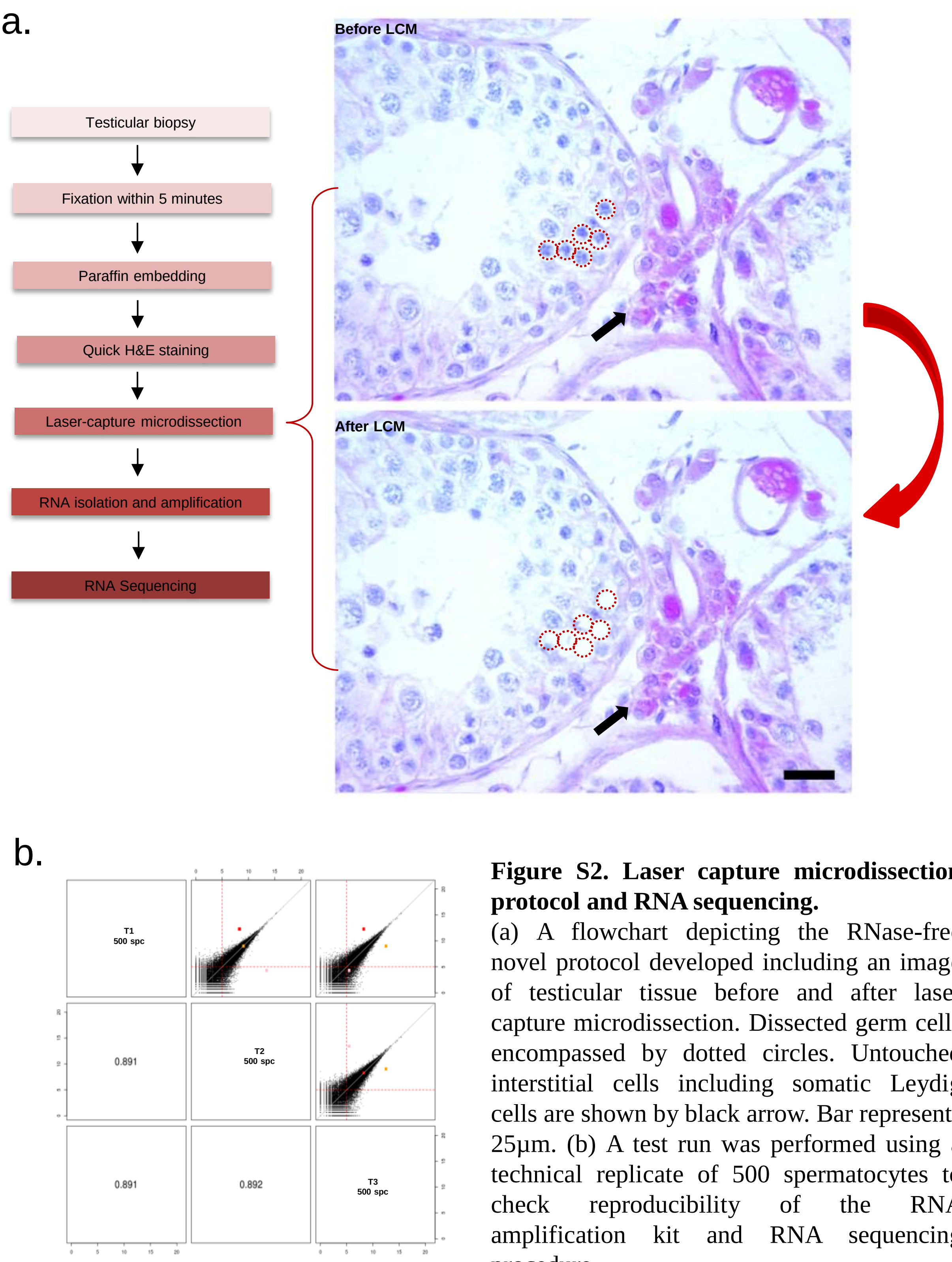

Figure S2. Laser capture microdissection protocol and RNA sequencing.
(a) A flowchart depicting the RNase-free novel protocol developed including an image of testicular tissue before and after laser capture microdissection. Dissected germ cells encompassed by dotted circles. Untouched interstitial cells including somatic Leydig cells are shown by black arrow. Bar represents 25µm. (b) A test run was performed using a technical replicate of 500 spermatocytes to check reproducibility of the RNA amplification kit and RNA sequencing procedure.

Development • Supplementary information

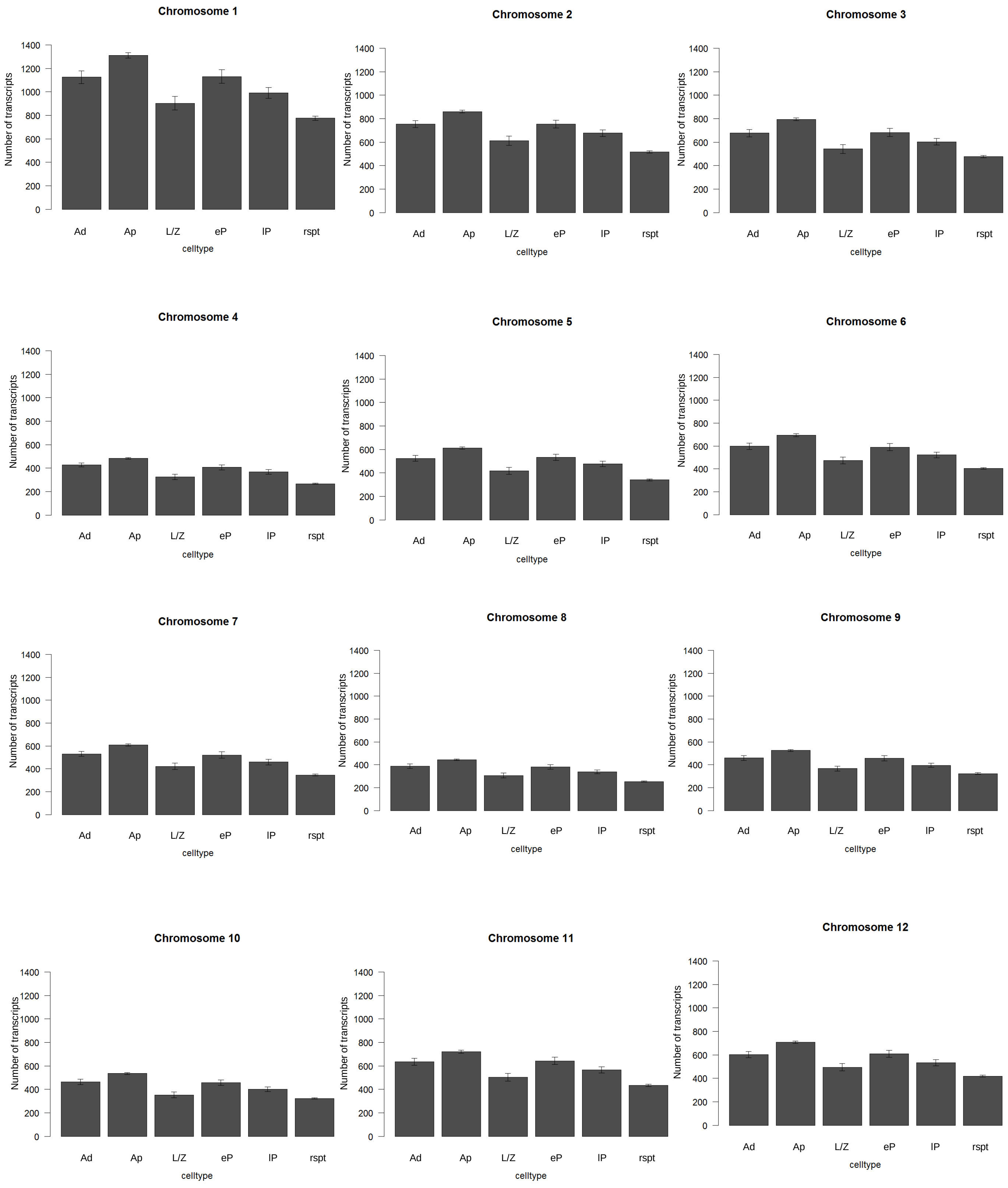

Development • Supplementary information

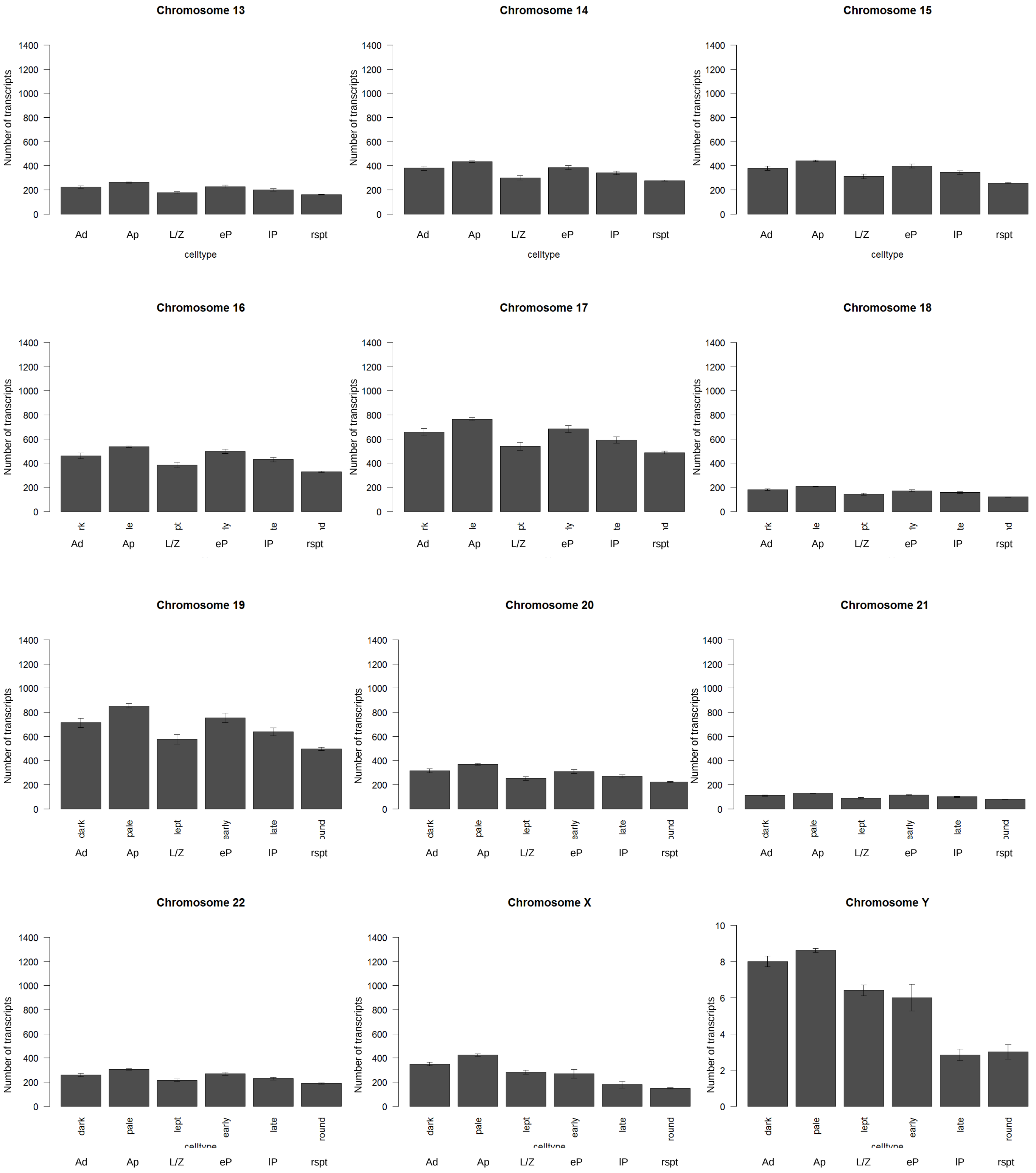

Figure S3. Gene expression distribution across chromosomes.
Graphs showing the distribution (mean ±SD) of the expressed transcripts across the chromosomes in each germ cell subtype. Based on counts per million. Ad; Adark and Ap; Apale spermatogonia, L/Z; leptotene/zygotene, eP; early pachytene and lP; late pachytene spermatocytes, rspt; round spermatids.

Development • Supplementary information

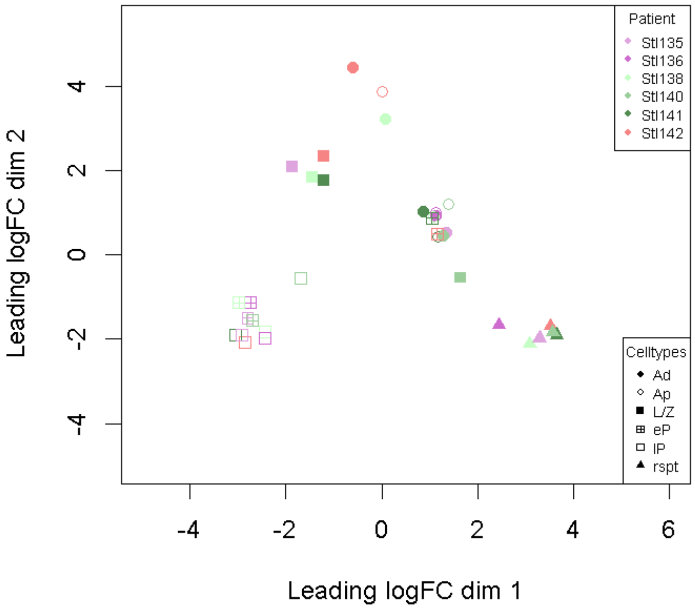

Figure S4. MDS plot between germ cell subtypes

MDS plot displaying the variation between germ cell subtypes including sample information. Symbols represent germ cell subtypes: filled circle: A_{dark} (Ad) spermatogonia, open circle: A_{pale} (Ap) spermatogonia, filled square: leptoeten/zygotene (L/Z) spermatocyte, crossed square: early pachytene (eP) spermatocyte, open square: late pachytene (lP) spermatocyte, filled triangle: round spermatids (rspt). Colors represent the origin of testis samples in the experiment.

Development • Supplementary information

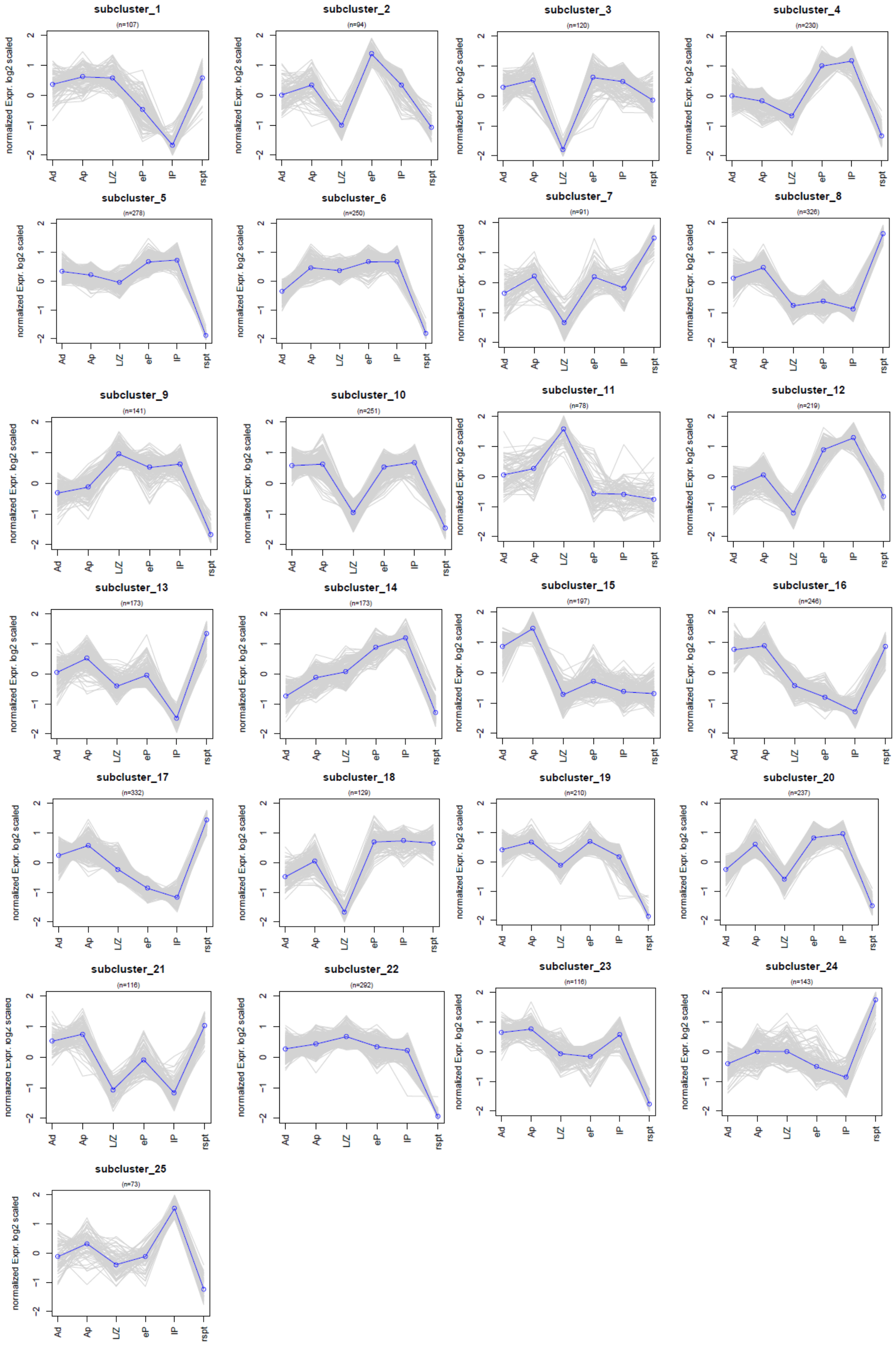

Figure S5. K-means plots of differentially expressed genes
K-means plots (25x) were used to categorize the 4622 genes that were differentially expressed between successive germ cell subtypes.

Development • Supplementary information

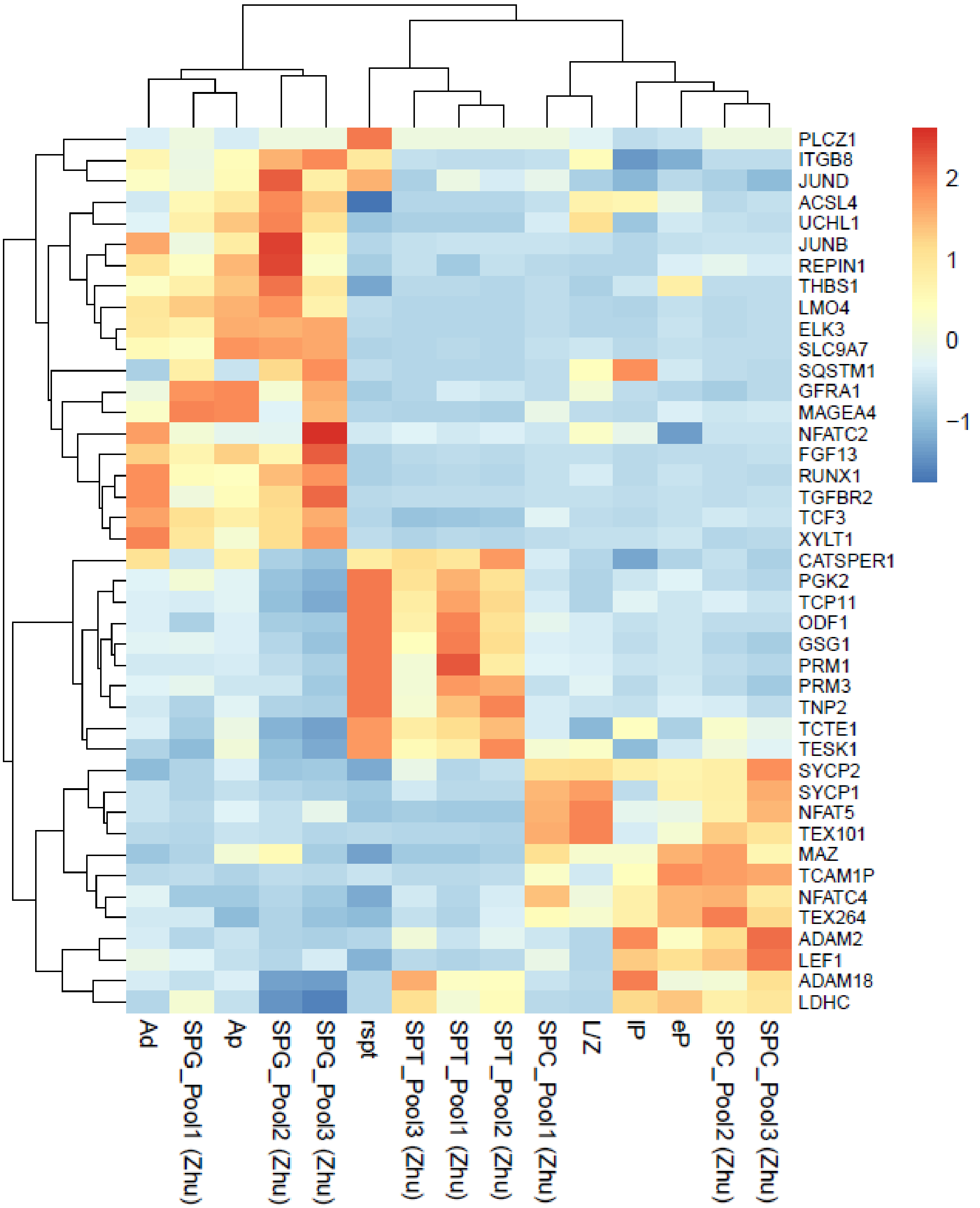

Figure S6. Clustered heatmap of selected gene set.
Clustering of the previously described gene set (Zhu et al. 2016) (figure 3c) for germ cell types with that of the spermatogenic phases and germ cell subtypes in this study.

Development • Supplementary information

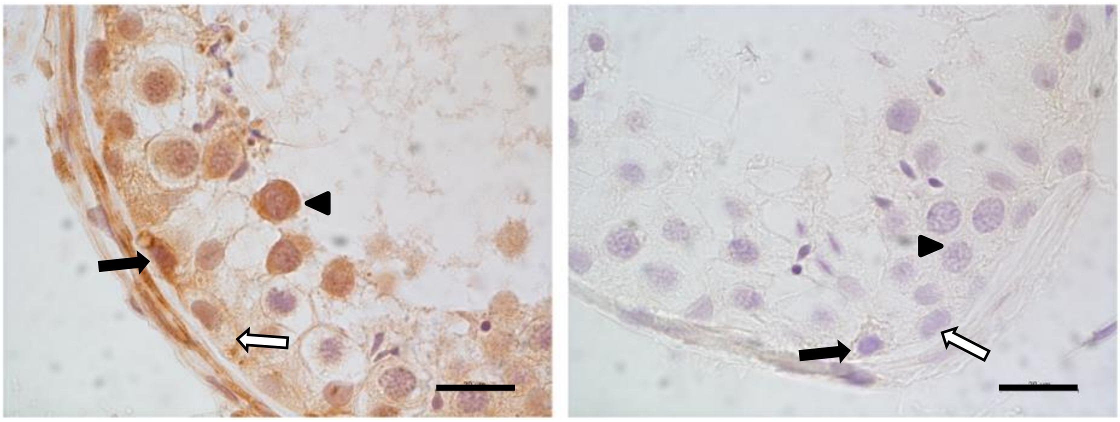

Figure S7. Immunohistochemistry of TPH1 expression in human testis section.
Validation of potential differences in expression of *TPH* between A dark and A pale spermatogonia on the protein level. Left panel:TPH is expressed in A dark spermatogonia (black arrow) and late pachytene spermatocytes (black arrowhead), but to a lesser extent in A pale spermatogonia (open arrow). This indicate a difference in TPH expression between A dark and A pale spermatogonia. Right panel: negative control. Bar represents 20μm

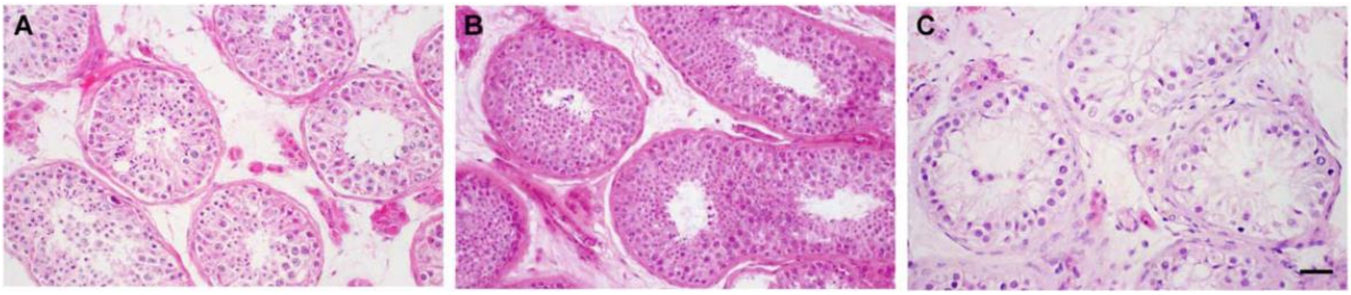

Figure S8. Histology of testis tissue used for RT-PCR.
(a) and (b) hematoxylin and eosin staining of testis tissue from men with complete spermatogenesis: Stl141 and Stl142, respectively, containing all germ cell subtypes and somatic cells. (c) hematoxylin and eosin staining of testis tissue from a man suffering from spermatogonial arrest: URO0074, containing only spermatogonia and somatic cells. Bar: 40µM.

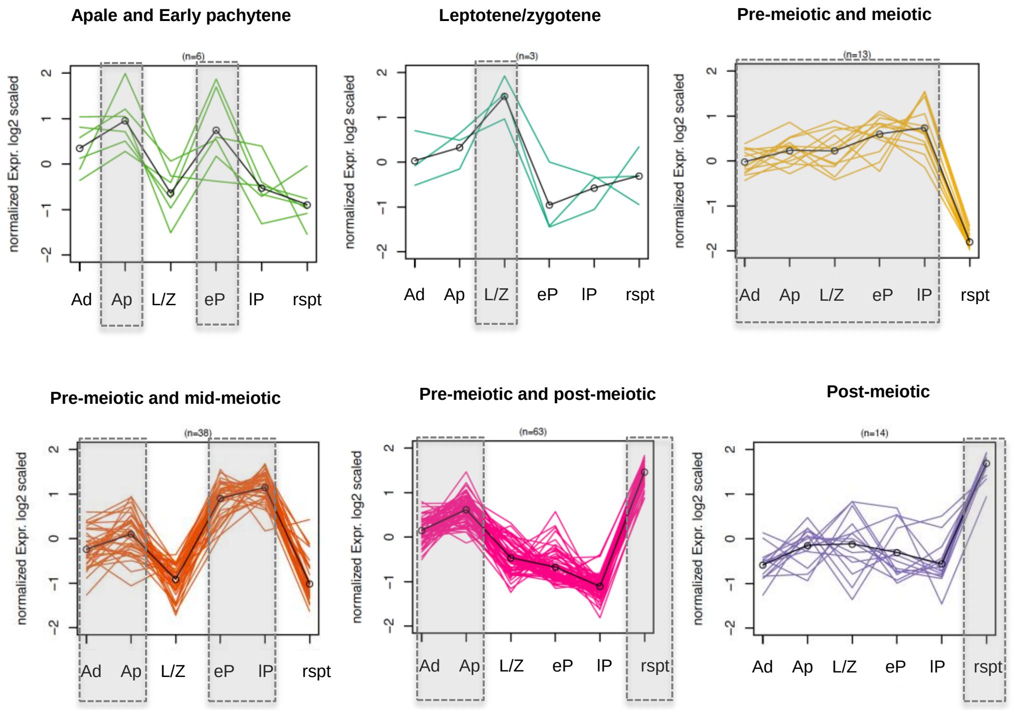

Figure S9. Expression patterns of differentially expressed long non-coding RNAs.
K-means clusters corresponding to the 137 differentially expressed lncRNAs. Areas of relatively high expression are indicated by grey boxes. Ad; Adark and Ap; Apale spermatogonia, L/Z; leptotene/zygotene, eP; early pachytene and lP; late pachytene spermatocytes, rspt; round spermatids.

Development • Supplementary information

Table S1

Click here to Download Table S1

Table S2

Click here to Download Table S2

Table S3

Click here to Download Table S3

Table S4

Click here to Download Table S4

Table S5

Click here to Download Table S5

Table S6

Click here to Download Table S6

Development • Supplementary information

Table S7

Click here to Download Table S7

Table S8

Click here to Download Table S8

Table S9

Click here to Download Table S9

Development • Supplementary information
